# *Tet1* is not required for myeloid leukemogenesis by *MLL-ENL* in novel mouse models

**DOI:** 10.1371/journal.pone.0248425

**Published:** 2021-03-11

**Authors:** Ryoichi Ono, Masahiro Masuya, Naokazu Inoue, Makoto Shinmei, Satomi Ishii, Yuri Maegawa, Bishnu Devi Maharjan, Naoyuki Katayama, Tetsuya Nosaka

**Affiliations:** 1 Department of Microbiology and Molecular Genetics, Mie University Graduate School of Medicine, Tsu, Mie, Japan; 2 Department of Hematology and Oncology, Mie University Graduate School of Medicine, Tsu, Mie, Japan; 3 Research Institute for Microbial Diseases, Osaka University, Osaka, Japan; Beth Israel Deaconess Medical Center-Harvard Medical School, UNITED STATES

## Abstract

The *Ten Eleven Translocation 1* (*TET1*) gene encodes an epigenetic modifying molecule that is involved in demethylation of 5-methylcytosine. In hematological malignancies, loss-of-function mutations of *TET2*, which is one of the *TET* family genes including *TET1*, are frequently found, while the mutations of *TET1* are not. However, clinical studies have revealed that *TET1* is highly expressed in some cases of the hematological malignancies including acute myeloid leukemia. Indeed, studies by mouse models using conventional *Tet1* knockout mice demonstrated that *Tet1* is involved in myeloid leukemogenesis by *Mixed Lineage Leukemia* (*MLL)* fusion gene or *TET2* mutant. Meanwhile, the other study showed that *Tet1* is highly expressed in hematopoietic stem cells (HSCs), and that deletion of *Tet1* in HSCs enhances potential self-renewal capacity, which is potentially associated with myeloid leukemogenesis. To examine the role of *Tet1* in myeloid leukemogenesis more precisely, we generated novel conditional *Tet1*-knockout mice, which were used to generate the compound mutant mice by crossing with the inducible *MLL-ENL* transgenic mice that we developed previously. The leukemic immortalization *in vitro* was not critically affected by conditional ablation of *Tet1* in HSCs with the induced expression of *MLL-ENL* or in hematopoietic progenitor cells retrovirally transduced with *MLL-ENL*. In addition, the leukemic phenotypes caused by the induced expression of *MLL-ENL in vivo* was not also critically affected in the compound mutant mouse model by conditional ablation of *Tet1*, although we found that the expression of *Evi1*, which is one of critical target genes of *MLL* fusion gene, in tumor cells was remarkably low under *Tet1*-ablated condition. These results revealed that *Tet1* was dispensable for the myeloid leukemogenesis by *MLL-ENL*, suggesting that the therapeutic application of Tet1 inhibition may need careful assessment.

## Introduction

Emerging evidence has demonstrated critical roles of epigenetic modifying molecules in oncogenesis [1]. Several findings over these decades suggest that cancer stemness is associated with aberrant epigenetic pathways caused by oncogenic driver mutations, including mutation of epigenetic modifier genes [2,3], similarly to the finding that stemness of embryonic stem cells (ESCs) is closely associated with epigenetic pathways [4].

The *Ten Eleven Translocation 1* (*TET1*) gene, which was originally identified as a fusion partner of *Mixed Lineage Leukemia* (*MLL*) in acute myeloid leukemia (AML) [5,6], encodes an epigenetic modifying molecule [7]. Tet1 has a catalytic dioxygenase domain consisting of a cysteine (Cys)-rich region and a double-stranded β-helix (DSBH) at the C-terminal part [8]. The dioxygenase is capable of converting 5-methylcytosine (5-mC) to 5-hydroxymethyl-C (5-hmC) and catalyzing further stepwise oxidation of the derivatives [9]. Tet1 belongs to the Tet family, including Tet2 and Tet3, which is characteristic of conserved Cys-rich and DSBH motifs [8,9].

Previous studies revealed that loss-of-function mutations of *TET2* are frequently found in hematological malignancies, while the mutations of *TET1* are not [10]. *Tet2* deficiency in mice recapitulated myeloproliferative disease (MPD) and lymphoma in humans [11]. In contrast, *Tet1* deficiency did not lead to MPD, but developed lymphoma with a long latency [12,13]. Meanwhile, the conventional knockout/knockdown of *Tet1* suppressed leukemic transformation by *MLL-AF9* through the downregulation of critical downstream molecules [14]. In addition, *Tet1* deficiency suppressed a *Tet2*-loss-driven myeloid malignancy, but promoted the lymphoid malignancy [15]. In line with the results, high *TET1* expression is found to be associated with a poor prognosis in cytogenetically normal AML [16]. These results suggested that *TET1* is a tumor-suppressor gene in lymphoid malignancies, but an oncogene in myeloid malignancies.

Several studies have demonstrated that, at least in part, normal hematopoietic stem cells (HSCs) share molecular mechanism with leukemic stem/initiating cells and ESCs [17]. Interestingly, *Tet1* is relatively highly expressed in HSCs and ESCs in comparison with their progenitor cells [7,13], suggesting the possible essential role of Tet1 in these stem cells. Also, a report suggested an important role of Tet1 in reprogramming [18]. However, *Tet1* deficiency in ESCs did not abrogate pluripotency [12], and on the contrary, the deletion in HSCs enhances potential self-renewal capacity [13], reminiscent of the deletion of *Tet2*. HSCs are considered to be one of major target cells of driver mutation(s) that initiate AML [19]. Thus, a concept of *Tet1* as an oncogene in myeloid malignancies might be contradictory in the leukemogenesis arising in HSCs, but this hypothesis remains to be investigated.

To clarify the role of *Tet1* in myeloid leukemogenesis more closely, we have established a new mouse model with conditional ablation of *Tet1* and induction of *MLL-ENL* in bone marrow (BM) cells. Using this model, we demonstrate that leukemic transformation by *MLL-ENL in vivo* and *in vitro* was not critically affected by the *Tet1*-ablation, although the expression of *Evi1*, which is one of critical target genes of *MLL* fusion gene, in tumor cells was remarkably low under *Tet1*-ablated condition. Also, leukemic immortalization by retroviral transduction of *MLL-ENL in vitro* was not inhibited by the conditional *Tet1*-ablation. Our results suggest that the therapeutic application of Tet1 inhibition in AML may need careful assessment.

## Materials and methods

### Reagents

4-hydroxytamoxifen (4-OHT) was dissolved in ethanol, and used at a final concentration of 0.1 μM. Tamoxifen (Sigma-Aldrich, St. Louis, MO) was dissolved in ethanol (100 mg/ml), and diluted in corn oil to 10 mg/ml. Starting at 8–12 weeks of age, or 5 weeks after bone marrow transplantation (BMT), tamoxifen was injected intraperitoneally at a dose of 100 mg/kg for 3 consecutive days. The time course started with the date of the third treatment as day 0. For drug selection in retroviral transduction, G418 (Thermo Fisher Scientific Inc., Waltham, MA) was used at a final concentration of 1 mg/ml.

### Mice

**Fig 1:**
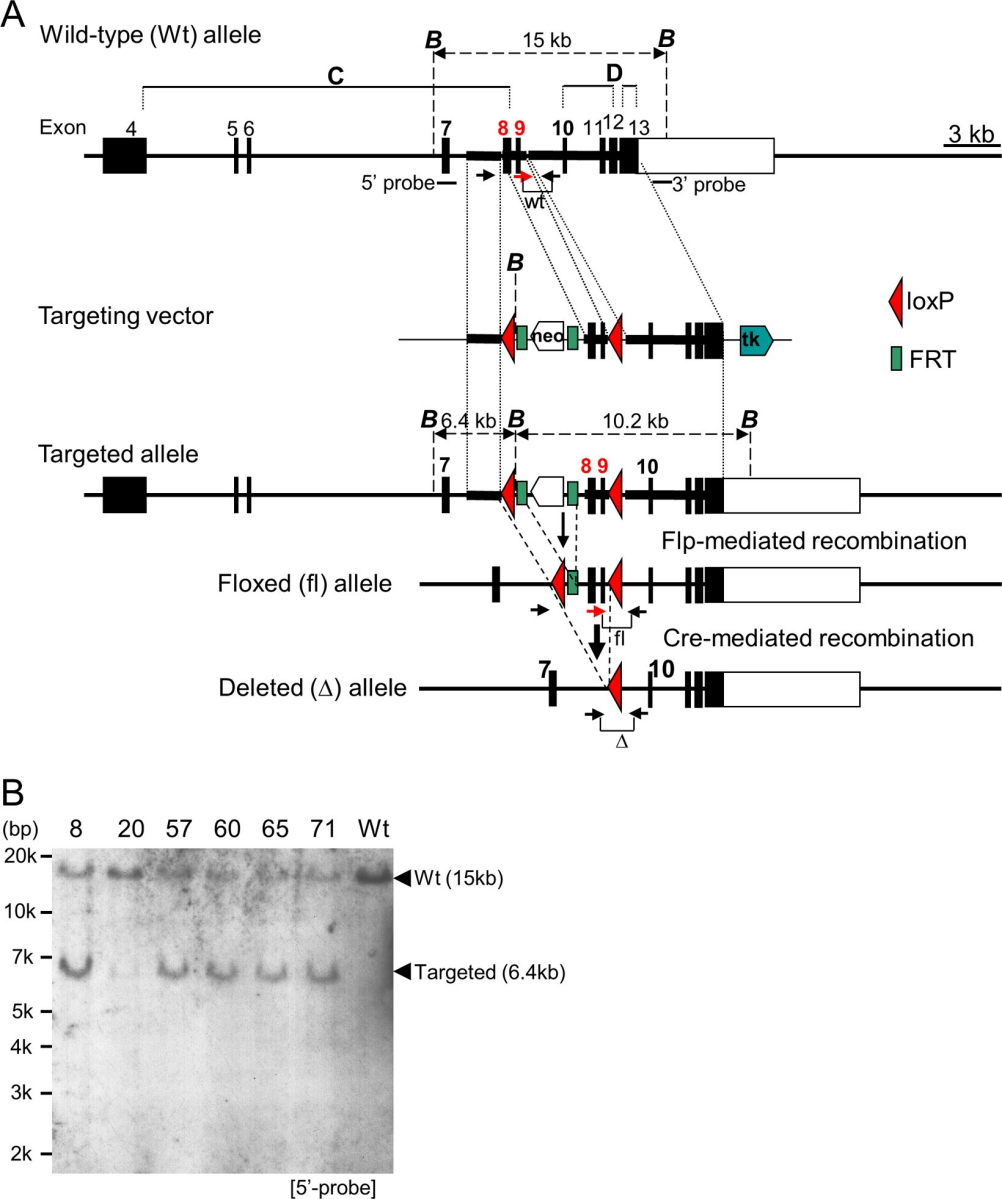
Generation of *Tet1*^fl/fl^ mice. (A) Schematic illustration of the targeting vector and the wild-type (wt) and genetically engineered *Tet1* alleles. The targeting vector was constructed by inserting 3 fragments (indicated by horizontal bold lines) corresponding to the wt regions (as shown), before the proximal loxP, between both loxP, and after distal loxP sequences, into the backbone vector. To generate the floxed (fl) allele, the *neomycin resistance* gene (*neo*) cassette flanked by the flippase (Flp) recognition target (FRT) sequences in the targeted allele was removed by mating with *Flpe* mice. The floxed allele can be recombinated between loxP sequences by Cre activity, resulting in the deleted (Δ) allele lacking exons 8 and 9. The restriction sites of *Bam*H I (*B*), and the 5’ and 3’ probes, used for Southern blot analysis, are indicated with vertical dashed lines and short horizontal lines, respectively. Coding and non-coding exons are indicated by closed and open boxes, respectively. The exons coding a cysteine-rich (*C*) and a double-stranded β-helix (*D*) regions are shown. *tk*, *thymidine kinase* gene. (B) Southern blot analysis of ES clones. Each genomic DNA extracted from 6 candidate ES clones and wt ES cells was digested with *Bam*H I and hybridized with the 5’ probe, resulting in the wt 15-kb and targeted 6.4-kb bands. The blot image was cropped from S1 Raw images.

C57BL/6N (B6) mice harboring a floxed allele (*Tet1*^fl/+^) where loxP sites flank exons 8 and 9 of *Tet1* were produced with standard techniques of gene targeting of B6 ES cells and flippase (Flp)-mediated recombination, as described, with some modifications [20]. Briefly, to generate *Tet1*^fl/fl^ mice, a targeting vector to introduce the 5’ and 3’ lox P sites, upstream of exon 8 and downstream of exon 9, respectively, was constructed on the pNT1.1 backbone vector (kindly provided by Dr. M. Okabe, Fig 1A). A 1.7-kb genomic fragment upstream of exon 8, a 1.1-kb genomic fragment covering exon 8, intron 8, and exon 9, and a 6.1-kb genomic fragment downstream of exon 9 were generated by polymerase chain reaction (PCR) of DNA from RP24-174M19 BAC clone (BACPAC Resources Center, Oakland, CA) using Phusion High-Fidelity DNA Polymerase (NEB, Ipswich, MA) according to the manufacturer’s protocol. These three fragments were inserted upstream of the proximal loxP site ligated with the *neo* cassette flanked by Flp recognition target (FRT) sites, between the *neo* cassette and the distal loxP, and between the distal loxP and the *thymidine kinase* (*tk*) cassette, respectively, in the pNT1.1. The primer sequences were as follows: 5’-GAAACTGCAGCCTATCATGCTGTGCTGACC-3’ and 5’-GAAAGACTCGAGGATTAAAGGCGTGCGCCAC-3’, 5’-GAAAGAATTCCTAGCACACGGAAGGCAGA-3’ and 5’-GTTTGTACGCGTCAGAGAAAGAGATTCATACATGCAATTAGGTG-3’, and 5’-CACGTCAGAGATGGTGGGTTGCAAACATGTCCACTTGTC-3’ and 5’-GAAACATCTAGAGAAGCCTTTAGACCCAACGATTGTAGGGTCCCGCAAC-3’, respectively (underlined nucleotides are added for subcloning). Each subcloned plasmid was validated by sequencing.

The targeting vector was linearized by *Not* I digestion just upstream of the insertion site of the 1.7-kb fragment, and electroporated into B6 EGR-101 male ES cells, followed by positive and negative selection with G418 and ganciclovir, respectively, as previously described [20]. To detect the correct homologous recombination, PCR screening of genomic DNA from the engineered ES cells was performed using LA Taq (Takara Bio Inc., Otsu, Japan) according to the manufacturer’s protocol. The primer sequences were as follows: 5’-GGCCTATCATGCTGTGCTGACCCTTTTGGGCT-3’ and 5’- GCTCCCGATTCGCAGCGCATCGCCTTCTAT-3’ for the first screening, and 5’- CTGCTAAAGCGCATGCTCCAGACTGCCTTGGGAAAAG-3’ (first PCR) or 5’- GAAAAGCGCCTCCCCTACCCGGTAGAATTGACCT-3’ (second PCR), and 5’- CCCACTACACCACATTAGCAAAGGCATTACATGAGAGAAGCCT-3’ for the second screening. To validate the screening, the karyotypes of PCR-positive clones were analyzed, and Southern blot analysis of genomic DNA from the clones with the correct karyotype was performed.

Chimeric male mice (F0), which were generated by the blastocyst injection of properly targeted ES cell clones, were bred with B6 wild-type female mice. F1 mice that were heterozygous for the targeted allele on a pure B6 background were bred with *Flp*e deleter mice (kindly provided by Dr. A. F. Stewart) to remove the FRT-flanked *neo* cassette. F2 mice that were heterozygous for the modified allele harboring the proximal loxP-FRT and the distal loxP sequences, as well as *Flp*e, were bred with B6 wild-type mice, to produce *Tet1*^fl/+^ mice that were heterozygous for the floxed allele of *Tet1* alone, and *Tet1*^fl/fl^ mice were generated by intercrossing of the *Tet1*^fl/+^ mice.

C57BL/6-*Gt(ROSA)26Sor*^*tm9(Cre/ESR1)Arte*^ (*CreER*) mice were purchased from Taconic Biosciences (Rensselaer, NY), and inducible *MLL-ENL* transgenic, Tg-*ME* (*ME*), mice harboring the cytomegalovirus early enhancer/chicken β actin (CAG) promoter-driven loxP-enhanced green fluorescent protein (*EGFP*)-poly A-loxP-*MLL-ENL*-poly A-loxP cassette were described [21].

The genotyping of mice or cells harboring a floxed/recombinated allele of *Tet1* and a *CreER* allele was performed by PCR of genomic DNA using LA Taq with the following individual 3 primer sets: dFRT-S2 (5’-GGGTTGCTGGTAAGTTTGAGGCT-3’), L-FS2 (5’-GTGGGTTGCAAACATGTCCACTTGT-3’), and L-dP-AS2 (5’-GAGATCAGAAACACACCTTTTCTGGTG-3’) for *Tet1*, and R26-S (5’-CTTCCCTCGTGATCTGCAACTC-3’), R26-AS1 (5’-CCGAGGCGGATCACAAGCAAT-3’), and Cre-1AS (5’-CAAACGGACAGAAGCATTTTCCAGGT-3’) for *CreER*. Genotyping of each offspring of Tg-*ME* mice expressing EGFP were performed by a fluorescence activated cell sorting (FACS) analysis of peripheral blood (PB) samples, and the recombinated allele was detected by PCR as previously described [21].

All animal studies were approved by the Animal Care Committees of Mie University (No. 24–34). Mice were kept in the specific pathogen-free facility under standard conditions (temperature 22–24°C; 12-h light/12-h dark cycle, and free access to food and water). In BMT experiments, recipient mice were housed in sterilized cages with filter-cap, and were administered neomycin (1 mg/ml (Merck Millipore, Burlington, MA)) in drinking water, for 4 weeks after BMT. Both male and female mice were subjected to experiments, except for that female mice were used for recipients in BMT. Animal health was monitored carefully and regularly in all experiments by trained staff. Hematological analyses of conditionally *Tet1*-ablated mice were performed, compared with the corresponding littermate controls, as previously described [22]. The mice were euthanized by carbon dioxide (CO_2_) inhalation, to analyze or collect BM cells. The total number of mice used in this study was 108.

### Purification of mouse hematopoietic stem and/or progenitor cells

Mouse c-Kit(+), c-Kitc^high^Sca-1^high^Lineage^-^ (KSL), CD34(-/+) KSL, c-Kit^high^Sca-1^-^Lineage^-^ (hereafter designated as MP) cells were purified from BM cells using FACS Aria (BD Biosciences, Franklin Lakes, NJ) as described, with some modifications [21]. In brief, BM mononuclear cells (BMMNCs) were prepared from 10- to 15-week-old B6 mice (n = 1–2 per each experiment). In myeloid immortalization assays and BMT assays, non-littermate mice with the indicated genotypes were used, since it was difficult to obtain a pair of littermates with four types of genotypes (described later). Using a MACS cell separation system (Miltenyi Biotec, Auburn, CA), c-Kit(+)-BM cells were enriched from BMMNCs labeled with biotinylated anti-c-Kit antibody (2B8), or lineage-depleted cells were isolated from BMMNCs labeled with Lineage Cell Detection Cocktail-Biotin (Miltenyi Biotec). The lineage-depleted cells were stained with streptoavidin (SAV)-peridinin chlorophyll protein (PerCP)-Cy5.5, and Alexa Fluor 647-conjugated anti-CD34 (RAM34), phycoerythrin (PE)-conjugated anti-c-Kit (2B8), and PE-Cy7-conjugated anti-Sca-1 (D7) monoclonal antibodies. KSL, CD34(-/+) KSL, and MP cells were purified from the stained cells using a FACS Aria II (BD Biosciences). All monoclonal antibodies were purchased from Biolegend (San Diego, CA), with the exception of the anti-CD34 antibody (Thermo Fisher Scientific Inc.).

### Retroviral transduction

The pMYs retroviral vector harboring *MLL-ENL*-internal ribosomal entry site (IRES)-*neo* was previously described [21]. Retroviral transduction was performed using RetroNectin (Takara Bio Inc.) with retroviral supernatants, which were harvested 48 hours after transfection from Plat E cells transfected with a retroviral construct, as previously described [23,24].

### Myeloid immortalization assay

Myeloid immortalization assays using serial replating were performed as described [21]. Briefly, the purified CD34(-) or CD34(+) KSL (150) and MP (1500) cells were directly sorted into 1.5 ml of methylcellulose medium prepared from M3234 (StemCell Technologies, Vancouver, Canada) according to the manufacture’s protocol, supplemented with 25 ng/ml SCF, 10 ng/ml each of IL-6, mouse IL-3, mouse GM-CSF (Miltenyi Biotec), and 0.1 μM of 4-OHT. The sorted cells in 1 ml of the mixture were immediately plated in 35 mm dishes. Alternatively, retrovirally transduced KSL, CD34(+) KSL, and MP cells were plated in the same M3234-based medium supplemented with G418 in addition to the cytokines. After culturing for 5 (fo MP cells), 6 (for CD34(+) KSL cells), and 10 days (for retrovirally transduced cells and CD34(-) KSL cells), colonies were enumerated, and single-cell suspensions (0.3–1×10^4^ cells) of colonies were subsequently replated in α-MEM-based medium containing 1% methylcellulose (Shin-Etsu Chemical Co., Ltd., Tokyo, Japan) supplemented with only the same cytokines. Every 5–7 days, replating of cells collected from colonies was repeated in the same way using the α-MEM-based methylcellulose medium. The immortalized cells were harvested from colonies in the third plating, and were cultured in α-MEM supplemented with 20% FBS and the same cytokines.

### BMT and leukemogenesis assay

Hematopoietic reconstitution of BM and leukemogenesis assays using BMT using B6 mice aged 10–15 weeks were performed as previously described [21], with some modifications. Briefly, for BM chimera mice, 1×10^6^ BM cells derived from the tested genotype of mice that were positive for CD45.2 were transplanted into lethally irradiated (7.5 Gy) wild-type recipient B6 mice that were positive for CD45.1. The recipient mice were periodically monitored, and analyzed morphologically and immunophenotpically at the indicated intervals as previously described [24].

For the mixed-BM chimera mice for use in leukemogenesis assays, a mixture of 1×10^6^ BM cells each derived from wild-type CD45.1-positive mice and the tested genotype of CD45.2-positive mice were also transplanted into wild-type CD45.1- (BMT #1 (#1)), or CD45.1/CD45.2- (BMT #2 (#2)) positive recipient female B6 mice that were lethally irradiated in the same way. Mixed-BM chimera mice were treated with tamoxifen after engraftment. For secondary transplantation, 3×10^3^ MPD cells harvested from BM were transplanted into sublethally irradiated (5.25 Gy) recipient B6 mice. To assess the mixed-BM chimera mice hematologically, PB samples collected from tail vein were measured 5 weeks after BMT to confirm the engraftment of transplanted cells, and also every 4 weeks after the treatment of tamoxifen. Mouse health was monitored for signs of illness such as less activity, scruffiness, weight loss, and hind-limb paralysis every day. The mice showing these signs were considered moribund, and were immediately euthanized by CO_2_ inhalation with all efforts to minimize animal suffering (n = 11 (#1), 13 (#2), and 7 (secondary BMT)), however, some mice unexpectedly died without showing suspicious signs of illness (n = 10 (#1), 6 (#2), and 6 (secondary BMT)). The control mice in #1 were euthanized in the same way at the end of monitoring (n = 9). The monitoring was ended after all the mice transplanted with Tg-*ME* cells met the criteria of humane endpoints described above. The analyses of BM and spleens from the tested mice were performed as previously described [24], showing the development of lethal MPD in the mice transplanted with Tg-*ME* cells.

### Immunophenotyping by FACS analysis

Immunophenotypic analyses were performed using a FACS Calibur or a FACS Canto II (BD Biosciences) as previously described [21]. Briefly, red blood cells in PB, BM, and spleen samples were lyzed with Ammonium-Chloride-Potassium lysis buffer beforehand. For the assessment of conditional *Tet1*-knockout mice, PB cells were stained with fluorescein isothiocyanate (FITC)-conjugated anti-CD11b (M1/70), PE-conjugated anti-CD3ε (145-2C11), PerCP-Cy5.5-conjugated anti-Gr-1 (RB6-8C5), and APC-conjugated anti-B220 (RA3-6B2) monoclonal antibodies. BM cells were labeled with Lineage Cell Detection Cocktail-Biotin, followed by staining with SAV-PerCP-Cy5.5, FITC-conjugated anti-CD34 (RAM34 (BD Biosciences)), PE-conjugated Sca-1 (D7), and APC-conjugated anti-c-Kit antibodies (2B8; for CD34^-/+^ KSL), or SAV-FITC, PE-conjugated anti-IL-7 receptor α (A7R34), PE-Cy7-conjugated anti-Sca-1, and APC-conjugated anti-c-Kit antibodies (for CLP). For MEP, GMP, and CMP, BM cells were labeled with Lineage Cell Detection Cocktail-Biotin and biotinylated anti-Sca-1 antibody (E13-161.7), followed by staining with SAV-PerCP-Cy5.5, FITC-conjugated anti-CD34, PE-conjugated anti-CD16/32 (93), and APC-conjugated anti-cKit antibodies. For the assessment of colony-forming cells, the cells were stained with FITC-conjugated anti-CD11b (M1/70), PE-conjugated anti-c-Kit, PerCP-Cy5.5-conjugated anti-Gr-1 (RB6-8C5), Alexa Fluor 647-conjugated anti-CD34, and PE-Cy7-conjugated anti-Sca-1 antibodies. For the assessment of cells from BMT recipient mice, chimerism in PB and BM cells was analyzed by staining with FITC-conjugated anti-CD45.2 (104) and PerCP-Cy5.5-conjugated anti-CD45.1 (A20) antibodies. The BM cells were stained with FITC-conjugated anti-CD11b, PerCP-Cy5.5-conjugated anti-Gr-1, and APC-conjugated anti-B220 antibodies, or PE-conjugated Sca-1, PerCP-Cy5.5-conjugated anti-Gr-1, and APC-conjugated anti-cKit antibodies. With the exception of the anti-CD34 antibodies, all monoclonal antibodies were purchased from BioLegend. Data were analyzed with FlowJo version 7.2.5 (BD Biosciences).

### Dot blot assay

Dot blot assay was performed as previously described [25], with some modifications. Briefly, Genomic DNA was manually spotted onto Hybond-N+ membranes (Cytiva, Marlborough, MA). After ultraviolet crosslinking, the membranes were probed with anti-5-hmC rabbit polyclonal (Active Motif, Carlsbad, CA), and anti-5-mC mouse monoclonal (33D3, Active Motif) antibodies, followed by probe with horseradish peroxidase (HRP)-conjugated anti-rabbit/anti-mouse immunoglobulin G (IgG) polyclonal antibodies (MBL, Nagoya, Japan), respectively. Detection was performed using Western Blotting Luminol Reagent (SANTA CRUZ BIOTECHNOLOGY, INC., Dallas, TX). The dot blot intensity was quantified by ImageJ software (National Institute of Health, version 1.53e, http://rsb.info.nih.gov/ij/download.html).

### Southern blot analysis

*Bam*H I-digested genomic DNA was analyzed as previously described [20], with some modifications. Briefly, blotted membranes were probed with DIG-labeled DNA probes, and visualized with CDP-star, using DIG-High Prime DNA Labeling and Detection Starter Kit I (Sigma-Aldrich), according to the manufacturer’s protocol. The template DNA for 5’ probe or 3’ probe was generated by PCR of genomic DNA from wild-type ES cells with LA Taq using the following primers: 5’-GTCTGGCTCCTGATGTATAAAGCTTG-3’ and 5’-GTCCTGCTACTCACAGGCTAGT-3’, and 5’-GTCTTCTGAAGTCACCTCGTCAATTAACT-3’ and 5’-GGCAGAACACTGGAACACAAAGTAATC-3’.

### RNA extraction and reverse transcription (RT)

Total RNA was extracted from cells using TRI Reagent LS (Molecular Research Center Inc., Cincinnati, OH), followed by treatment with RQ1 RNase-free DNase I (Promega, Madison, WI). RT was performed using SuperScript II (Thermo Fisher Scientific Inc.) with random hexamers, as previously described [24].

### Northern blot analysis

Poly-A^+^ RNA (0.5 μg) purified from total RNA using an Oligotex™ -dT30<Super> mRNA Purification Kit (Takara Bio Inc.) was analyzed as previously described [5], with some modifications. Briefly, blotted membranes were probed with DIG-labeled RNA probes synthesized using T3 (for *Actb*) or T7 (for *Tet1*) RNA polymerase and DIG RNA Labeling Mix (Sigma-Aldrich) supplemented with RNAse inhibitor (recombinant RNasin (Promega)), in DIG Easy Hyb solution (Sigma-Aldrich), according to the manufacturer’s protocol. Detection was performed using CDP-star in the same way as done in Southern blot analysis. The corresponding sequences of probes were as follows: *Actb*, 112–909; *Tet1* exons 8–9, 4518–4764; and *Tet1* exons 10–13, 4883–6120.

### PCR

To analyze the exon structure of *Tet1* transcripts around the region flanked by lox P sequences by RT-PCR, PCR of cDNA was performed using LA Taq with primers spanning exons 7–11 (5’- TTGAGGTCCTACAGCGGACAT-3’ and 5’- ACGCCCCTCTTCATTTCCA-3’), as previously described [24]. RT-quantitative PCR (qPCR) was performed using PowerSYBR® Green PCR Master Mix on a StepOnePlus™ Real-Time PCR System (Applied Biosystems, Foster City, CA), as described [21]. After quantifying the expression levels of the samples using the 2^-ΔΔCT^ method and normalization relative to *B2m*, the relative expression levels were calculated. The sequences of the primers used in qPCR, with the exception of those for *B2m*, *MLL-ENL*, *Hoxa9*, *Meis1a*, *Evi1*, and *Dnmt3b* [21], were as follows: 5’-CATCCCACAGACCGAAGATGTAC-3’ and 5’-TTTTGGGTCGATGCCTTGAC-3’ for *Tet1*, 5’-TTGGGCAAGTGTGGCATATG-3’ and 5’-TCTCACCCTGACAACAACAGTTTC-3’ for *Tet2*, 5’-ACAGCCTGCATGGACTTCTGT-3’ and 5’-ACGCAGCGATTGTCTTCCTT-3’ for *Tet3*, 5’-AGAGATGCCATCACCCAAAAA-3’ and 5’-ATCGATGCTCACCTTCTGATAGTAAG-3’ for *Dnmt1*, and 5’-GGCATCCACTGTGAATGATAAGC-3’ and 5’-GTGGTAATGGTCCTCACTTTGCT-3’ for *Dnmt3a*.

### Statistical analysis

Unpaired Student’s *t*-tests, and a one-way analysis of variance (ANOVA) followed by Tukey-Kramer test as a post hoc test, were used to compare two groups, and more than two groups, respectively. A two-way repeated measures ANOVA followed by Shaffer’s modified sequentially rejective Bonferroni procedure as post hoc analyses was used to compare several groups at different timepoints. Data from RT-qPCR were log2-transformed before the statistical analysis. To compare the gene expression from CD34(-) KSL-derived cells, after a one-way ANOVA was used to compare four genotypes of samples at the first round of plating and a two-way ANOVA was used to compare two pairs of two genotypes samples at the first and third rounds of plating were performed, the obtained p values were adjusted using the Holm-Bonferroni correction. The probability of overall survival of the mice was estimated using the Kaplan-Meier method and compared by the log-rank test. All statistical tests were performed using the R software program (version 3.5.2, https://www.r-project.org/), except that two-way repeated measures ANOVA tests were performed using the ANOVAKUN software (version 4.8.4, http://riseki.php.xdomain.jp/index.php?ANOVA%E5%90%9B), which was executed on the R software program.

## Results

### Generation of conditional *Tet1*-knockout mice

To conditionally ablate *Tet1 in vivo*, we designed a strategy to induce Cre/loxP-mediated excision of the exons 8 and 9 of *Tet1*, which led to a truncated product lacking the catalytic domain by a frameshift mutation (Fig 1A). The targeting vector was constructed, and the targeted ES clones (Fig 1B) were used to generate chimeric founder mice, from which two lines (57 and 71) of F1 mice were obtained. After removing the *neomycin*^r^ cassette by *Flpe* deleter mice, *Tet1*^fl/+^ mice were established, and were interbred to generate *Tet1*^fl/fl^ (*T1*^fl/fl^) mice. The *CreER*;*Tet1*^fl/fl^ (*C*/*T1*^fl/fl^) mice were generated by mating *T1*^fl/fl^ and *CreER* mice.

### Validation of *Tet1*-ablation in hematopoiesis

**Fig 2:**
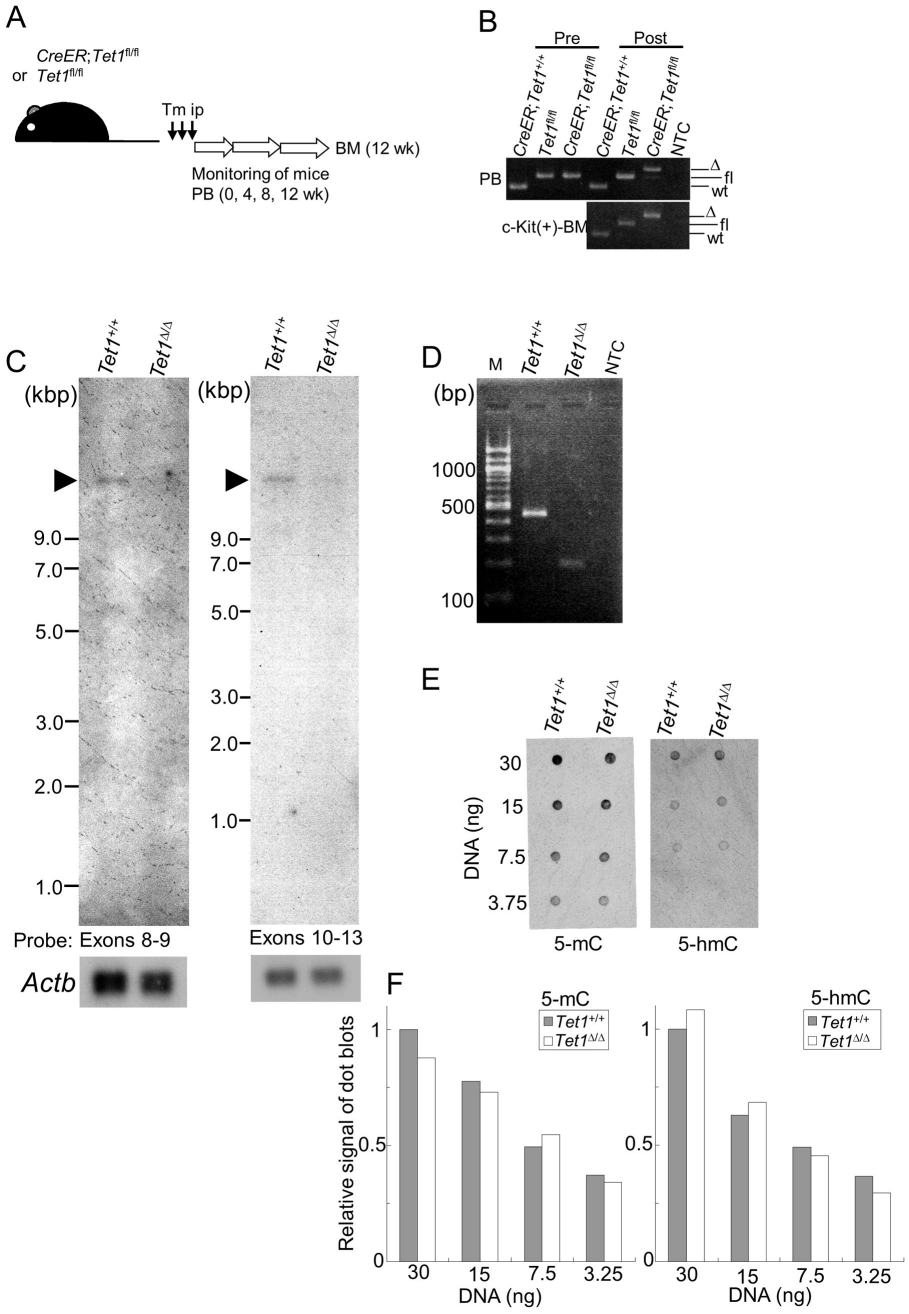
Conditional ablation of *Tet1 in vivo* and *in vitro*. (A) The experimental strategy for the hematological assessment of conditionally *Tet1*-ablated mice. Tm, tamoxifen; ip, intraperitoneal injection; PB, peripheral blood; BM, bone marrow. (B) Genomic PCR of PB and c-Kit(+)-BM cells just before (pre) and 4 weeks after (post) tamoxifen treatment using the primers shown in Fig 1A. *CreER*; *Tet1*^+/+^ mice were analyzed in the same way as controls. Bands derived from wild-type (wt), floxed (fl), and deleted (Δ) alleles at the *Tet1* locus are shown. (C) Northern blot analyses of CD34(-) KSL-derived cells immortalized by the induction of *MLL-ENL* with (*Tet1*^⊗/⊗^) or without (*Tet1*^+/+^) the simultaneous conditional ablation of *Tet1*, as shown in Fig 4A. Each poly-A^+^ RNA was hybridized with probes covering exons 8 and 9, and exons 10–13. Arrowheads indicate bands (approximately 12.7 kb) corresponding to the full-length transcript of *Tet1*. (D) RT-PCR of the immortalized cells with *Tet1*^Δ/Δ^ and *Tet1*^+/+^ using primers spanning exons 7–11. M, 100 bp ladder marker; NTC, negative control. (E) Dot blot assays of genomic DNA from the immortalized cells with *Tet1*^Δ/Δ^ and *Tet1*^+/+^. 5-hmC, 5-hydroxymethylcytosine; 5-mC, 5-methylcytosine. The blot and gel images were cropped from S1 and S2 Raw images.

First, to validate the genetic effect focused on hematopoiesis in *C*/*T1*^fl/fl^ mice, PB and c-Kit(+)-BM cells were analyzed in *C*/*T1*^fl/fl^ and *T1*^fl/fl^ mice treated with tamoxifen (Fig 2A). In these cells, efficient recombination in the floxed allele was detected at 4 weeks after treatment (Fig 2B). To examine the genetic effect more precisely, *MLL-ENL*-immortalized CD34(-)KSL cells highly expressing *Tet1* (described in detail later) were analyzed, since the high expression of *Tet1* is restricted to a small number of hematopoietic stem and immature progenitor cells. The *Tet1*^+/+^ immortalized cells expressed the full-length transcript of *Tet1* (an approximately 12.7-kb band, Fig 2C), whereas the *Tet1*^Δ/Δ^ immortalized cells did not. We also confirmed the expected alignment of exons in the *Tet1*^Δ/Δ^ immortalized cells by RT-PCR with primers spanning exons 7–11 (Fig 2D). However, dot blot assays of DNA from the immortalized cells revealed little significant alteration in the levels of 5-mC or 5-hmC by *Tet1*-ablation (Fig 2E and 2F), implying the limited involvement of Tet1 catalytic activity, and/or a redundant effect by Tet2 and/or Tet3. These results suggested that the Cre-mediated recombination was induced efficiently and correctly in hematopoietic cells, leading to genetic ablation of *Tet1*.

### Hematological assessment of conditionally *Tet1*-ablated mice

**Fig 3:**
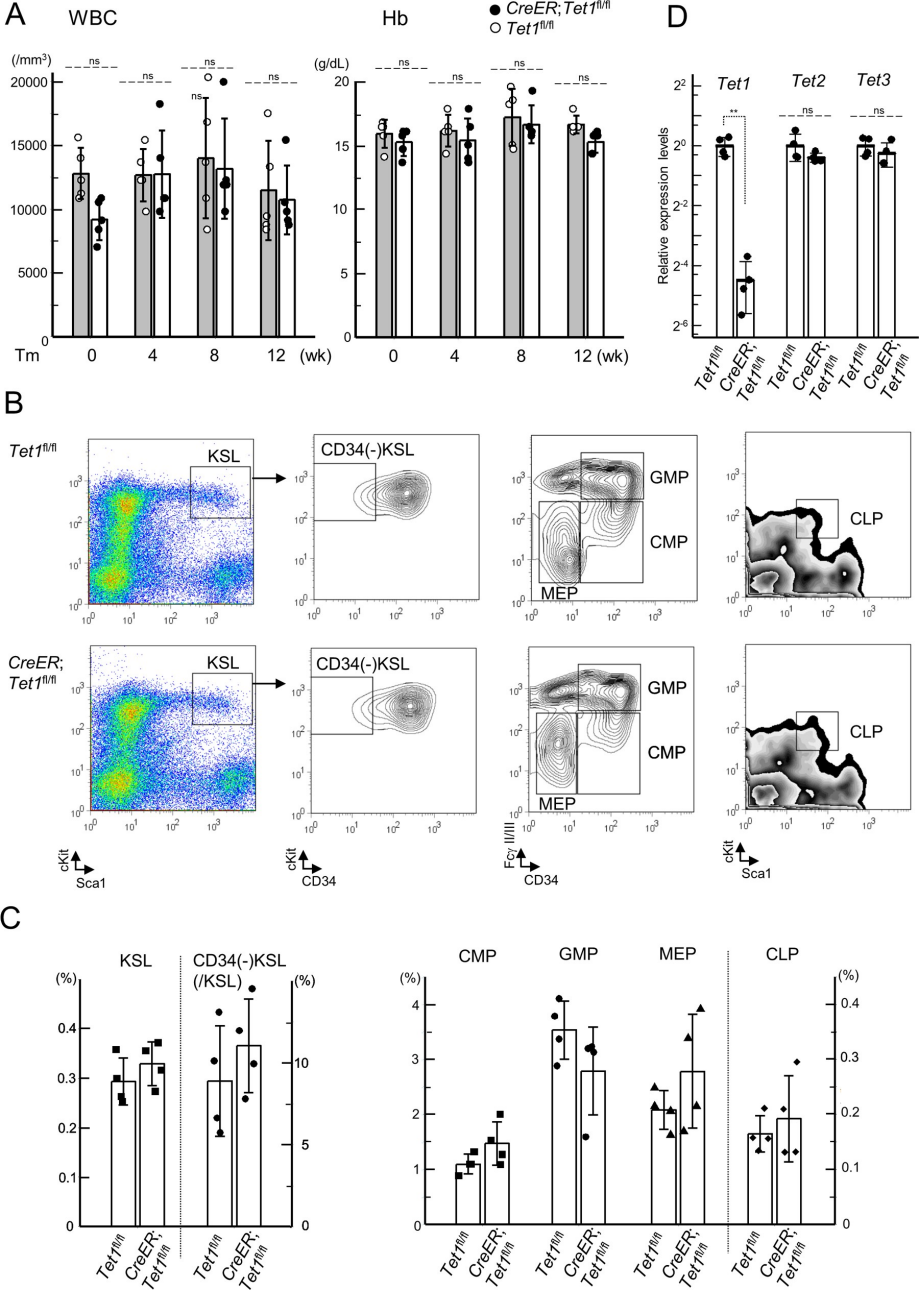
Hematological assessment of conditionally *Tet1*-ablated mice. (A) Measurement of the white blood cell (WBC) counts and hemoglobin (Hb) concentration in peripheral blood just before (0), 4, 8, and 12 weeks after tamoxifen treatment as shown in Fig 2A (n = 5 per each (total 10)). (B, C, and D) A FACS analysis of hematopoietic stem and progenitor cells (B and C), the expression levels of *Tet1*, *Te2*, and *Tet3* in bone marrow (BM) 12 weeks after tamoxifen treatment, determined by RT-qPCR (D). Representative FACS data of 4 independent experiments (with 1 pair per each (total 8)) are shown in (B). Bar graphs show the mean and SD of data combined from two (A) and four independent experiments (C and D), respectively. **p<0.005; n.s., not significant. (determined by two-way repeated measures ANOVA followed by Shaffer’s modified sequentially rejective Bonferroni procedure (A), and two-tailed unpaired *t*-tests (D), respectively).

We next assessed the biological effect of conditional *Tet1*-ablation on hematopoiesis. At the timepoints of 0 (just before the treatment), 4, 8, and 12 weeks after tamoxifen treatment, PB and BM (only 12 weeks) samples were collected from *C*/*T1*^fl/fl^ and *T1*^fl/fl^ mice (Fig 2A). However, no significant differences concerning white blood cells (WBCs) and hemoglobin (Hb) concentration in PB and the proportion of hematopoietic stem and progenitor cells (HSPCs) in BM were found (Figs 3A–3C and S1), while conditional *Tet1*-ablation did not affect the *Tet2* or *Tet3* expression (Fig 3D). In addition, we generated BM chimera mice, in which the BM cells of recipient mice were replaced with *C*/*T1*^fl/fl^ or *T1*^fl/fl^ BM cells using BMT (S2A Fig), and treated the chimera mice with tamoxifen after confirmation of engraftment. In this experimental system, it was assumed that the donor-derived HSCs were subjected to proliferative stress, which was partly associated with aging, in the process of reconstitution of hematopoiesis before the conditional ablation of *Tet1*. However, no significant differences concerning WBCs in PB were found within an observation period of 34 weeks (S2B Fig). Furthermore, no lethal disease in the tamoxifen-treated mice receiving *C*/*T1*^fl/fl^ BM cells. These results suggested that *Tet1* was dispensable in the maintenance of proper hematopoiesis, at least during the period of 34 weeks.

### A role of *Tet1* in leukemogenesis by *MLL-ENL* in HSPCs *in vitro*

**Fig 4:**
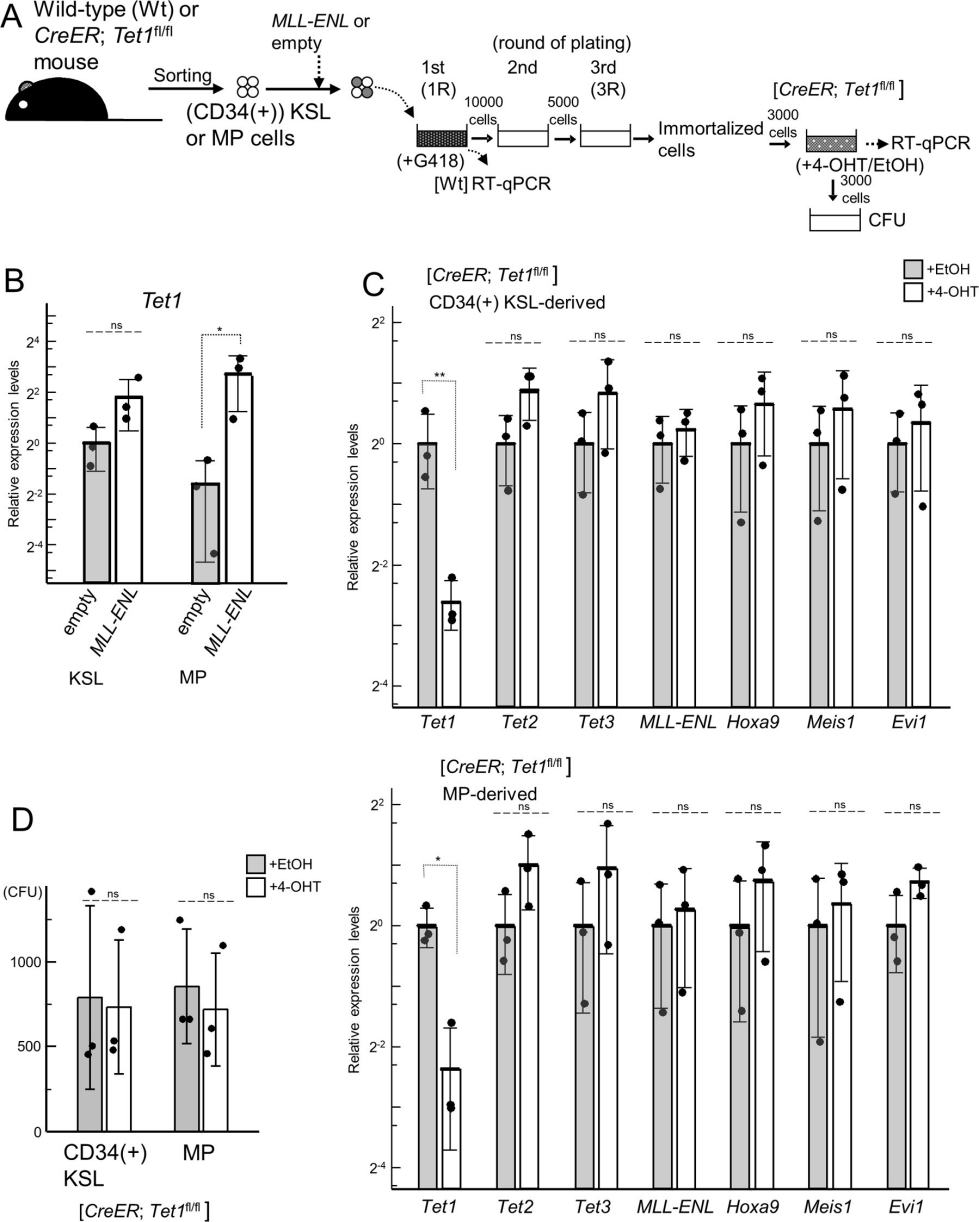
The conditional ablation of *Tet1* in cells retrovirally immortalized by *MLL-ENL*. (A) The experimental strategy for myeloid immortalization assays of the retrovirally transduced cells using colony replating, and conditional ablation of *Tet1* in the immortalized *CreER*;*Tet1*^fl/fl^ cells. Wild-type (Wt) KSL and MP cells (from one mouse per each (total 3)) were retrovirally transduced with *MLL-ENL* or an empty vector, respectively. *CreER*;*Tet1*^fl/fl^ CD34(+) KSL and MP cells were immortalized with the retroviral transduction of *MLL-ENL* after serial replating, and treated with 4-hidroxyl tamoxifen (4-OHT). (B) The expression level of *Tet1* in the wt *MLL-ENL*-transduced KSL and MP cells (from 2 mice) at the end of the first round of colony plating, as assessed by RT-qPCR. (C and D) The expression levels of *Tet1*, *Tet2*, *Tet3*, *MLL-ENL*, *Hoxa9*, *Meis1*, and *Evi1*, assessed by RT-qPCR (C) and clonogenicities (D) in the *CreER*;*Tet1*^fl/fl^ CD34(+) KSL/MP-derived cells retrovirally immortalized by *MLL-ENL* with treatment of 4-OHT or vehicle control (ethanol, EtOH). CFU, colony forming unit. Bar graphs show the mean and SD of three independent experiments in (B), (C), and (D). **p<0.005; *p<0.05; n.s., not significant. (determined by two-tailed unpaired *t*-tests).

Based on the finding that *Tet1* is upregulated by *MLL-AF9* [14], we analyzed the expression levels of *Tet1* in retrovirally *MLL-ENL*-transduced KSL and MP cells (Fig 4A), and found similar increase of *Tet1* expression, which was significant only in MP cells (Fig 4B). Next, to examine whether *Tet1* might be required for the maintenance of leukemic immortalization, *C*/*T1*^fl/fl^ CD34(+) KSL and MP cells were immortalized by *MLL-ENL* (Fig 4A). CD34(-) KSL cells were not analyzed in these immortalization assays, due to difficulty in retroviral transduction of *MLL-ENL* into CD34(-) KSL cells, as previously described [21]. The 4-OHT treatment of the immortalized *C*/*T1*^fl/fl^ cells led to an approximately 80% reduction in *Tet1* expression (Fig 4C), but clonogenicities in the treated cells were not significantly different from those in the control cells (Fig 4D). We also found no significant changes in the expression levels of the critical genes downstream of *MLL-ENL* or the epigenetic regulator genes associated with DNA methylation, *Tet2*, *Tet3*, and *Dnmt* genes, between the treated and the control cells (Figs 4C and S3). These results suggested that *Tet1* was upregulated by MLL-ENL in HSPCs, but was not required for the maintenance of *MLL-ENL*-mediated immortalization in CD34(+) KSL and MP cells.

### A role of *Tet1* in leukemogenesis by *MLL-ENL* in LT-HSCs *in vitro*

In *ME* mice we established [21], induction of *MLL-ENL* led to leukemic transformation of CD34(-) KSL cells enriched for long-term HSCs (LT-HSCs), but not to the transformation of CD34(+) KSL and MP cells. To examine the role of *Tet1* in *MLL*-fusion-mediated leukemogenesis in LT-HSCs, we generated *CreER*;*Tet1*^+/+^;Tg-*ME* (*C*/*ME*) and *CreER*;*Tet1*^fl/fl^;Tg-*ME* (*C*/*T1*^fl/fl^/*ME*) mice by mating the individual genetically engineered mice.

**Fig 5:**
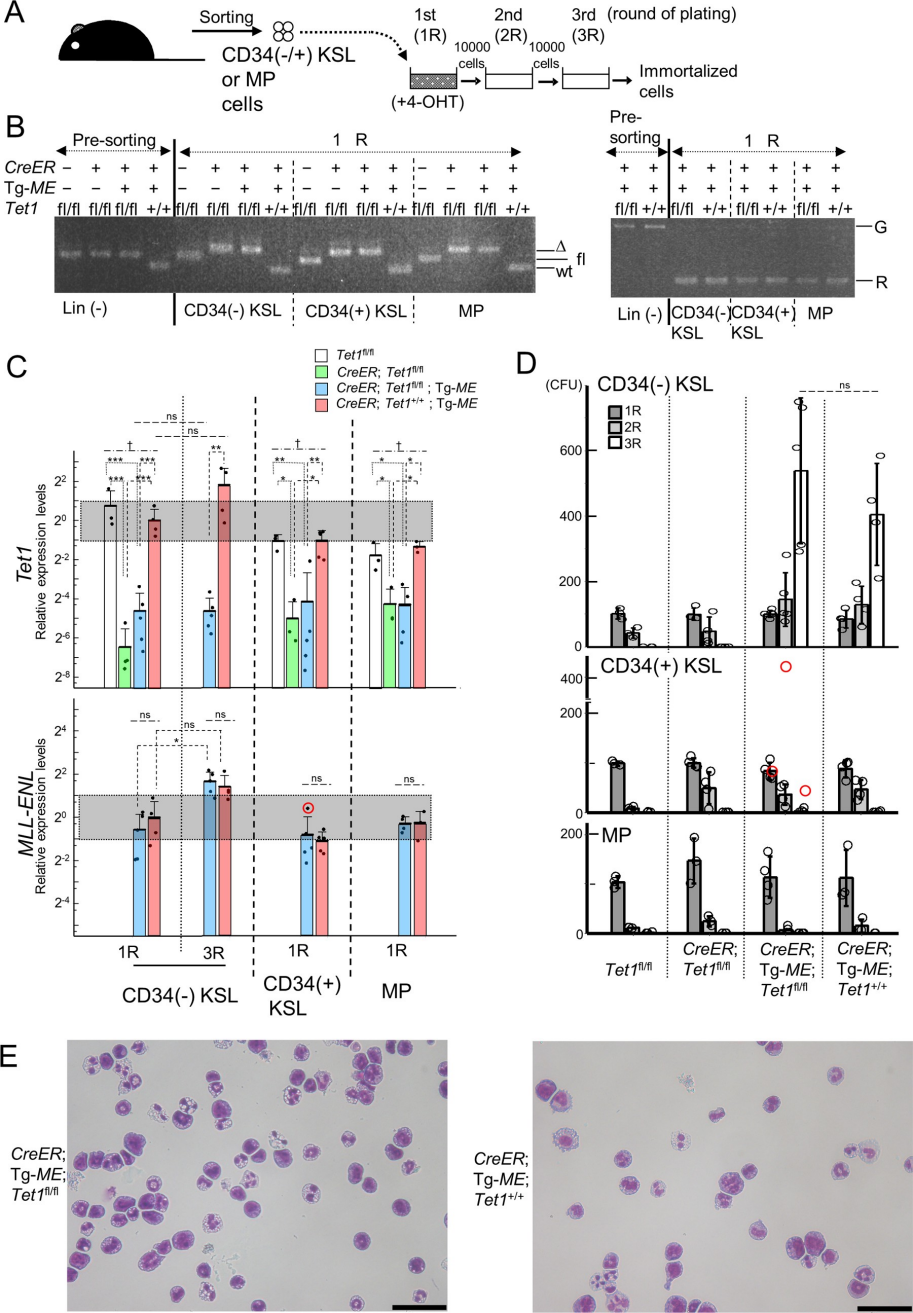
Leukemic immortalization by the induction of Tg-*ME*-derived *MLL-ENL* under simultaneous *Tet1*-ablation. (A) The experimental strategy for the myeloid immortalization assays of cells purified by sorting. CD34(-), CD34(+) KSL, and MP cells were purified from *Tet1*^fl/fl^, *CreER*; *Tet1*^fl/fl^, *CreER*; *Tet1*^fl/fl^; Tg-*ME*, and *CreER*; *Tet1*^+/+^; Tg-*ME* bone marrow cells (from one mouse per each genotype per each experiment (total 12 mice)), and were directly plated in the presence of 4-hidroxyl tamoxifen (4-OHT). (B) Genomic PCR of Lin-depleted (Lin (-))cells prepared for sorting (pre-sorting), and the cells harvested at the end of the first round (1R) of plating. Bands derived from wild-type (wt), floxed (fl), and deleted (Δ) alleles at *Tet1* locus (left panel), and those derived from germline (G) and recombinated (R) alleles regarding Tg-*ME* (right panel) are shown. (C) Expression levels of *Tet1* and *MLL-ENL* in the cells harvested at the end of the first (1R) and the third (3R, only in CD34(-) KSL-derived cells) rounds of plating, assessed by RT-qPCR. A red circle indicates one CD34(+) KSL sample with an exceptionally high *MLL-ENL* expression level. For *MLL-ENL*, p-values were determined by a two-way repeated measures ANOVA followed by Shaffer’s modified sequentially rejective Bonferroni procedure (CD34(-) KSL), and two-tailed unpaired *t*-tests (CD34(+) KSL and MP). (D) Colony numbers along serial replating in the myeloid immortalization assays. Red circles indicate colony forming units (CFUs) in the CD34(+) KSL sample with an exceptionally high *MLL-ENL* expression level, as shown in (C). *P* values were determined by two-tailed unpaired *t*-tests. (E) The typical morphology of the cells constituting the colonies derived from *CreER*; *Tet1*^fl/fl^; Tg-*ME* and *CreER*; *Tet1*^+/+^; Tg-*ME* CD34(-) KSL cells at the end of the third round of plating. Cells were stained with Wright-Giemsa. Magnification, 200×; scale bars, 50 μm. Bar graphs show the mean and SD of three independent experiments in (C) and (D). ***p<0.0005; **p<0.005; *p<0.05; n.s., not significant; †, not significant in comparison of the other combinations. With the exception of *MLL-ENL*, the p-values for the expression levels of the genes tested were determined by one-way ANOVA followed by Tukey-Kramer tests (CD34(+) and MP), or a combination of one-way ANOVA followed by Tukey-Kramer tests (1R) and two-way repeated measures ANOVA followed by Shaffer’s modified sequentially rejective Bonferroni procedure (*CreER*; *Tet1*^fl/fl^; Tg-*ME* and *CreER*; *Tet1*^+/+^; Tg-*ME*) adjusted using the Holm-Bonferroni correction (CD34(-) KSL). The gel images were cropped from S2 Raw images.

**Fig 6:**
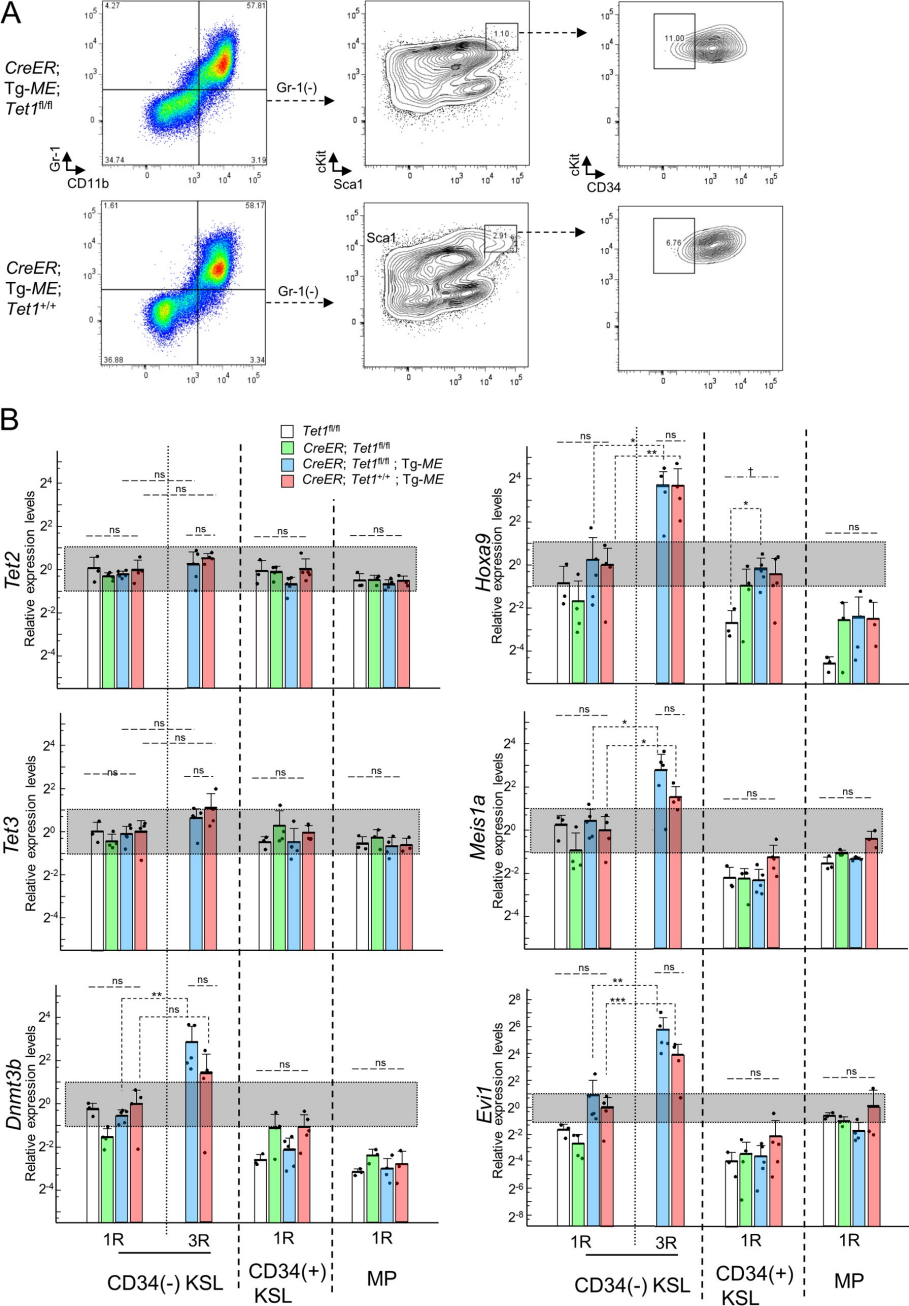
Characterization of hematopoietic stem/progenitor cells after the induction of Tg-*ME*-derived *MLL-ENL* under simultaneous *Tet1*-ablation. (A) The typical immunophenotype of the cells constituting the colonies derived from *CreER*; *Tet1*^fl/fl^; Tg-*ME* and *CreER*; *Tet1*^+/+^; Tg-*ME* CD34(-) KSL cells at the end of the third round of plating in Fig 5. (B) The expression levels of *Tet2*, *Tet3*, *Dnmt3b*, *Hoxa9*, *Meis1a*, and *Evi1* in the cells harvested at the end of the first (1R) and the third (3R, only in CD34(-) KSL-derived cells) rounds of plating in Fig 5, as assessed by RT-qPCR. Bar graphs show the mean and SD of three independent experiments in (B). ***p<0.0005; **p<0.005; *p<0.05; n.s., not significant; †, not significant in comparison of the other combinations. The p-values for the expression levels of the genes tested were determined by one-way ANOVA followed by Tukey-Kramer tests (CD34(+) and MP), or a combination of one-way ANOVA followed by Tukey-Kramer tests (1R) and two-way repeated measures ANOVA followed by Shaffer’s modified sequentially rejective Bonferroni procedure (*CreER*; *Tet1*^fl/fl^; Tg-*ME* and *CreER*; *Tet1*^+/+^; Tg-*ME*) adjusted using the Holm-Bonferroni correction (CD34(-) KSL).

We first performed myeloid immortalization assays of CD34(-) KSL, CD34(+) KSL, and MP cells from *T1*^fl/fl^, *C*/*T1*^fl/fl^, *C*/*T1*^fl/fl^/*ME*, and *C*/*ME* mice, respectively (Fig 5A). In the first plating, efficient recombination between loxP sites in *Tet1*^fl^ and/or Tg-*ME* alleles was confirmed (Fig 5B), accompanied by the similar reduction of *Tet1* and/or induction of *MLL-ENL* among the cells harboring *CreER*, and *Tet1*^fl/fl^ and/or Tg-*ME* (Fig 5C). Also, the four genotypes of CD34(-) KSL, CD34(+) KSL, and MP cells formed similar numbers of colonies, respectively. In the third plating, the induced *MLL-ENL* enabled *C*/*T1*^fl/fl^/*ME* and *C*/*ME* CD34(-) KSL-derived cells to form numerous highly proliferative colonies, leading to immortalization, without significant differences in the number of the colonies between the two genotypes (Fig 5D). Furthermore, both genotypes of the cells constituting the colonies exhibited similar morphological and immunophenotypic features of myeloid cells at various differentiation stages (Figs 5E and 6A). However, it should be noted that, in one of *C*/*T1*^fl/fl^/*ME* CD34(+) KSL samples, a relatively higher *MLL-ENL* expression enhanced clonogenicity temporally, but failed to immortalize after additional replating (Fig 5C and 5D).

Next, to examine whether the conditional *Tet1*-ablation may affect the molecular mechanism of leukemic immortalization by the induced *MLL-ENL*, we analyzed the critical genes downstream of *MLL-ENL* and the epigenetic regulator genes associated with DNA methylation, in the first and third (only for CD34(-) KSL) plating (Figs 6B and S4). In the first plating, the expression levels of these genes tested were not significantly changed among *T1*^fl/fl^-, *C*/*T1*^fl/fl^-, *C*/*T1*^fl/fl^/*ME*-, and *C*/*ME*-derived cells in each subpopulation, except for a small reduction of *Dnmt3a* in *C*/*T1*^fl/fl^- and *C*/*T1*^fl/fl^/*ME*-derived cells in CD34(-) KSL, respectively. In comparison of *C*/*T1*^fl/fl^/*ME* and *C*/*ME* CD34(-) KSL-derived cells in the third plating with those in the first plating, the *Tet1* expression was non-significantly enhanced in the third plating of *C*/*ME*-derived cells, while the *Tet1* expression did not increase with escape from the conditional ablation along serial plating in *C*/*T1*^fl/fl^/*ME*-derived cells. The *MLL-ENL* expression in the third plating was significantly enhanced in *C*/*T1*^fl/fl^/*ME*-derived cells, but was still almost comparable to that in *C*/*ME*-derived cells (Fig 5C). The expression levels of *Hoxa9*, *Meis1*, and *Evi1* in the third plating were significantly elevated in both genotypes, but were not significantly different between the two genotypes. Among the epigenetic genes tested, expression of *Dnmt3b* and the *Dnmt1* was significantly more strongly enhanced in the *C*/*T1*^fl/fl^/*ME*- and *C*/*ME*-derived cells, respectively, in comparison with each counterpart cells. Regarding *Dnmt3b*, our results were compatible with the finding in ES cells [26].

Taken together, these results indicated that the *Tet1*-ablation did not affect the leukemic immortalization by induced *MLL-ENL* in CD34(-) KSL, CD34(+) KSL, or MP cells.

### The phenotype of MPD by the induction of *MLL-ENL* was not affected by conditional ablation of *Tet1 in vivo*

**Fig 7:**
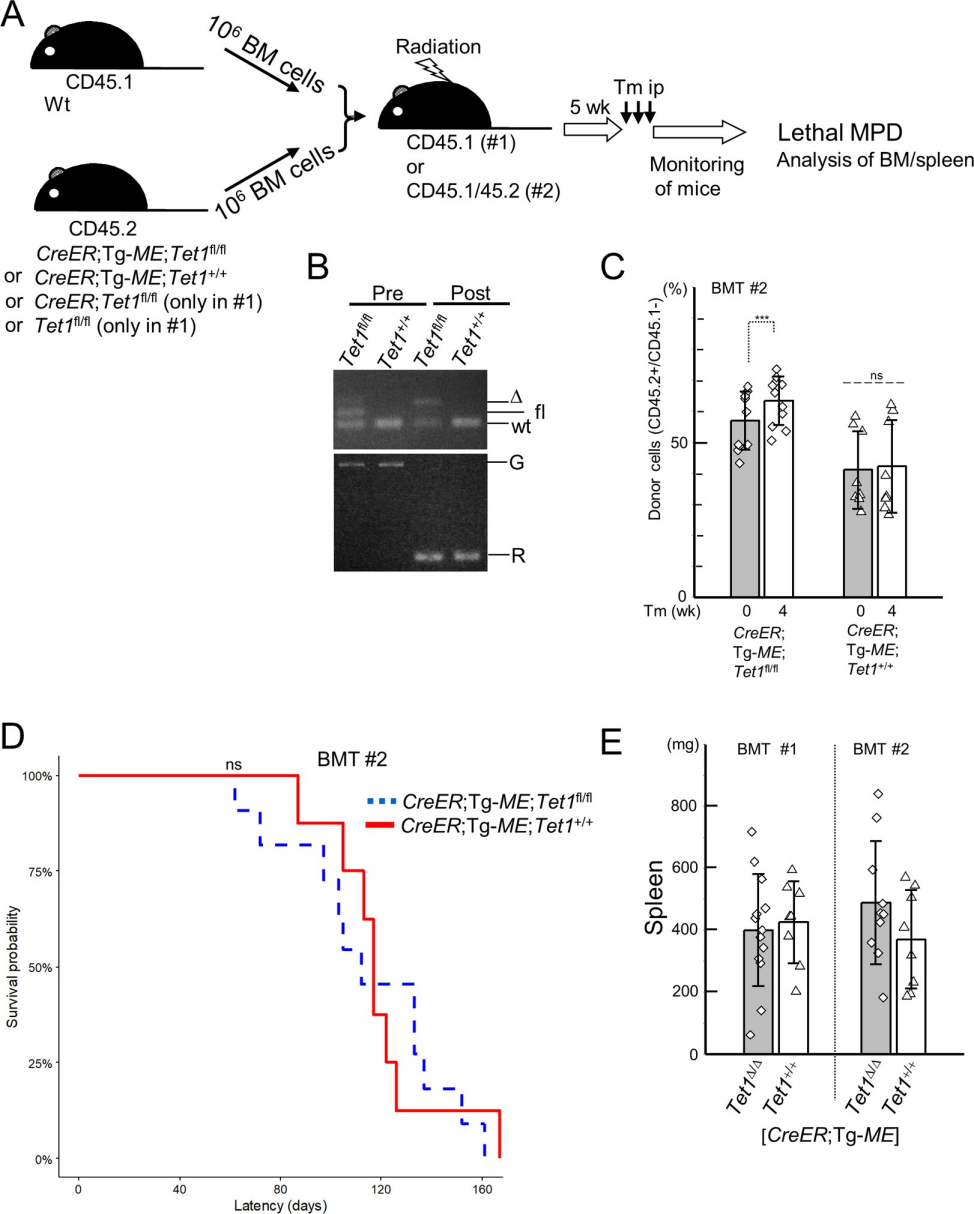
The leukemogenesis by induction of Tg-*ME*-derived *MLL-ENL* under simultaneous *Tet1* ablation *in vivo*. (A) The experimental strategy for leukemogenesis assays enabling leukemic initiation *in vivo*. The two types (BMT #1 and #2) of the mixed-bone marrow (BM) chimera mice (from one mouse per each genotype per each experiment (total 8 (#1) and 4 (#2)) were prepared using BMT into CD45.1-, and CD45.1/45.2-positive recipient mice, respectively. Wt, wild-type; Tm, tamoxifen; ip, intraperitoneal injection; MPD, myeloproliferative disease. (B) Genomic PCR of PB cells from the mixed-BM chimera mice receiving *CreER*; *Tet1*^fl/fl^; Tg-*ME* (*Tet1*^fl/fl^) and *CreER*; *Tet1*^+/+^; Tg-*ME* (*Tet1*^+/+^) BM cells, just before (pre) and 4 weeks after (post) tamoxifen treatment. Bands derived from wild-type (wt), floxed (fl), and deleted (Δ) alleles at *Tet1* locus (top panel), and those derived from germline (G) and recombinated (R) alleles regarding Tg-*ME* (bottom panel) are shown. (C) Chimerism analyses in peripheral blood cells just before (0) and 4 weeks after tamoxifen treatment in the cohort #2. *P*-values were determined by two-way repeated measures ANOVA followed by Shaffer’s modified sequentially rejective Bonferroni procedure. (D) Survival curves of the mixed-BM chimera mice (cohort #2) receiving *CreER*; *Tet1*^fl/fl^; Tg-*ME* (n = 11) and *CreER*; *Tet1*^+/+^; Tg-*ME* (n = 8) BM cells that were treated with tamoxifen. (E) Weight of the spleens from the moribund mice that developed lethal MPD. Data from two independent experiments were combined in (D). Bar graphs show the mean with SD of data combined from two independent experiments in (C) and (E). ***p<0.0005; n.s., not significant. (determined by two-way repeated measures ANOVA followed by Shaffer’s modified sequentially rejective Bonferroni procedure (C and E), and by log-rank tests (D), respectively). The gel images were cropped from S2 Raw images.

**Fig 8:**
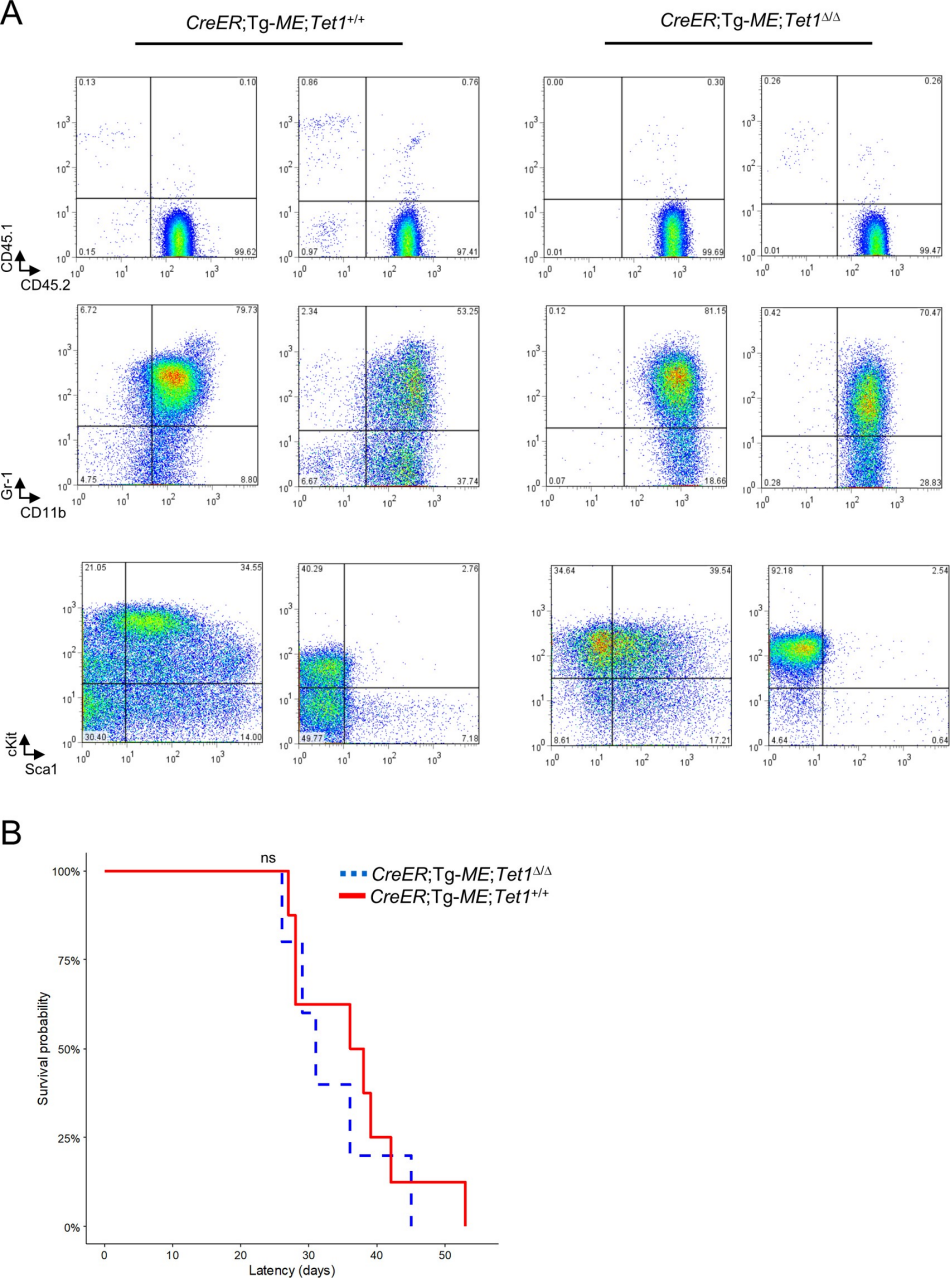
Characterization of tumor cells induced by Tg-*ME*-derived *MLL-ENL* under simultaneous *Tet1* ablation. (A) FACS analyses of bone marrow (BM) cells from the moribund mice that developed lethal MPD in Fig 7. (B) Survival curves of the secondarily transplanted mice that received primary MPD cells with *CreER*; *Tet1*^Δ/Δ^; Tg-*ME* (n = 5) and *CreER*; *Tet1*^+/+^; Tg-*ME* (n = 8) harvested from the respective BM. Data from two independent experiments were combined in (B). n.s., not significant. (determined by log-rank tests (B)).

To examine whether the conditional *Tet1*-ablation may affect leukemogenesis by induced *MLL-ENL in vivo*, *C*/*T1*^fl/fl^/*ME* and *C*/*ME* mice were preliminarily treated with tamoxifen, leading to lethal MPD. To avoid ubiquitous induction of *MLL-ENL*, we beforehand prepared two types (#1 and #2) of mixed-BM chimera mice by BMT of mixture of equal ratios of BM cells from *T1*^fl/fl^ (#1), *C*/*T1*^fl/fl^ (#1), *C*/*T1*^fl/fl^/*ME* (#1, #2), or *C*/*ME* (#1, #2) mice (CD45.2(+)), and wild-type mice (CD45.1(+)), into recipient mice (CD45.1(+) (#1) or CD45.1/CD45.2(+) (#2)) (Fig 7A). In PB cells collected 4 weeks after the treatment of tamoxifen, efficient recombination between loxP sites in *Tet1*^fl^ and/or Tg-*ME* alleles was confirmed (Fig 7B), while no significant changes of chimerism in each genotype were found, except for a little increase in the chimera mice (#2) receiving *C*/*T1*^fl/fl^/*ME* cells (Figs 7C and S5A). Over a period of about 200 days of observation after the treatment, the chimera mice receiving *C*/*T1*^fl/fl^/*ME* or *C*/*ME* cells died with similar latency in each cohort (#1 and #2), while the mice (#1) receiving *T1*^fl/fl^ or *C*/*T1*^fl/fl^ cells did not (Figs 7D and S5B). Almost comparable splenomegaly was found in the chimera mice receiving *C*/*T1*^fl/fl^/*ME* or *C*/*ME* cells, in each cohort (Fig 7E). Immunophenotyping analyses of BM cells from the moribund mice revealed that the majority of the cells derived from CD45.2-positive donor cells, and expressed myeloid markers and c-Kit, and that these cells also expressed markers of immaturity, c-Kit/Sca-1, in some recipient mice (Fig 8A), similarly in each cohort. These features were also compatible with the morphological findings of BM cells showing MPD where myeloid cells at various stages of differentiation occupied BM (S5C Fig). In addition, in secondary transplantation of BM cells from moribund mice, both genotypes of MPD were aggressively reproduced with similar latencies (Fig 8B).

### The *Evi1* expression was markedly lower in *CreER*;*Tet1*^Δ/Δ^;Tg-*ME* samples

**Fig 9:**
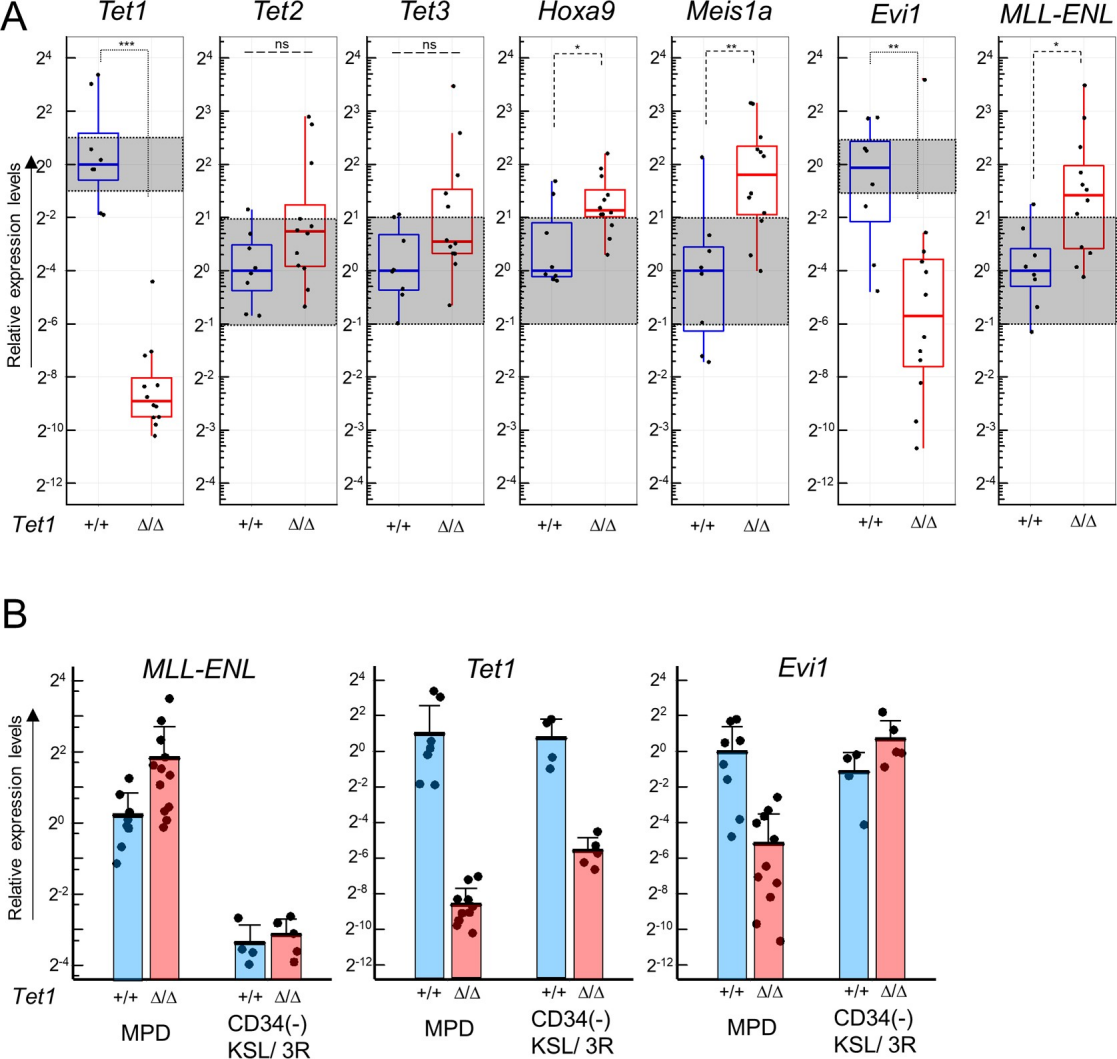
Expression profiling of *Tet* family genes and *MLL-ENL*-associated genes in the leukemogenesis assays. (A) The expression levels of *Tet1*, *Tet2*, *Tet3*, *Hoxa9*, *Meis1a*, *Evi1*, and *MLL-ENL* in BM cells from the moribund mice developing lethal MPD in the leukemogenesis assays under *Tet1*-ablated (*Tet1*^Δ/Δ^) and *Tet1*^+/+^-conditions, as assessed by RT-qPCR. Data are shown in box and whisker plots. ***p<0.0005; **p<0.005; *p<0.05; n.s., not significant. (determined by two-tailed unpaired *t*-tests). (B) The expression levels of *MLL-ENL*, *Tet1*, and *Evi1* in the MPD cells in comparison with those in the CD34 (-) KSL-derived cells expressing the induced *MLL-ENL* with (*Tet1*^+/+^) or without *Tet1* ablation (*Tet1*^Δ/Δ^) at the end of the third (3R) plating (shown in Fig 5A), as determined by RT-qPCR. Bar graphs show the mean with SD.

To further examine whether the conditional *Tet1*-ablation may affect the molecular mechanism of leukemogenesis by induced *MLL-ENL in vivo*, we analyzed the critical downstream genes and epigenetic genes in MPD cells from the moribund chimera mice (Fig 9A). The *Tet1* expression was significantly and remarkably lower in *C*/*T1*^Δ/Δ^/*ME* MPD cells. However, the expression levels of other epigenetic genes did not differ between the two genotypes of MPD cells (Figs 9A and S6). Unexpectedly, the *MLL-ENL* expression was mildly but significantly higher in *C*/*T1*^Δ/Δ^/*ME* MPD cells, accompanied with significantly higher expression levels of *Meis1* and *Hoxa9*. Nevertheless, in *C*/*T1*^Δ/Δ^/*ME* MPD cells, the *Evi1* expression was significantly and markedly lower (median: approximately 3%) in comparison with *C*/*T1*^+/+^/*ME* MPD cells, while it was exceptionally higher in one *C*/*T1*^Δ/Δ^/*ME* MPD sample. In comparison of the expression levels in the MPD samples with those of the third plating of CD34(-) KSL-derived cells in myeloid immortalization assays, the lower expression levels of *Evi1* in the *C*/*T1*^Δ/Δ^/*ME* MPD samples were highlighted, although those of *MLL-ENL* were higher in the MPD samples (Fig 9B).

Taken together, in the leukemogenesis by induced *MLL-ENL in vivo*, the conditional *Tet1*-ablation led to marked reduction of *Evi1* expression, but did not critically affect the MPD phenotype, although an unexpected increase of the *MLL-ENL* expression in *C*/*T1*^Δ/Δ^/*ME* MPD cells might cancel out the phenotypic change. Furthermore, *Tet1* was not involved in the maintenance of the leukemogenesis.

## Discussion

The present study redefines the role of *TET1*/*Tet1* in myeloid leukemogenesis by the *MLL*-fusion gene more closely. The aberrantly increased and reduced expression of *TET1* is found to be associated with myeloid and lymphoid leukemogenesis [13–15], respectively, although loss-of-function mutations of *TET2*/*Tet2* develops both leukemogenesis [11]. MLL-fusion protein upregulates the *TET1*/*Tet1* expression in the myeloid leukemogenesis [14], as confirmed in the present study. Interestingly, *TET1* is found to be crucial for the proliferation of human T-cell acute lymphoblastic leukemia, suggesting context-dependent roles of TET1/Tet1 in leukemogenesis [27]. Indeed, our results suggested that *Tet1* may not be necessarily essential for *MLL*-fusion-mediated myeloid leukemogenesis.

Our leukemogenesis assays *in vivo* showed that the conditional *Tet1*-ablation did not affect the myeloid leukemogenesis phenotype by induced expression of *MLL-ENL*. In comparison with BMT of retrovirally *MLL*-fusion-transduced HSPCs [14,17,22], our mixed-BM chimera enabled leukemic initiation to occur without temporary culture *in vitro*, thereby excluded the bias of engraftment and some inevitable influence by culture *in vitro*, as in conditional/inducible *MLL*-fusion-mediated mouse models [28,29]. Meanwhile, compared with conventional *Tet1* knockout mice [13–15], the Tet1 protein expression remained at the early phase of the conditional ablation. Thus, partly due to differences in the approaches, our results may differ from the finding [14] that Tet1 plays an essential role for the myeloid leukemogenesis.

*Tet1* was dispensable for the leukemic immortalization by *MLL-ENL* in CD34(-) KSL cells enriched for LT-HSCs, in which the role of *Tet1* had not been well-characterized, while, in retrovirally *MLL-AF9*-transduced Lin^-^ BM cells, *Tet1* is critically involved in the myeloid leukemogenesis [14]. Since the retroviral transduction into LT-HSCs is inferred to be very difficult [21], this discrepancy concerning the role of *Tet1* may reflect the difference of a subpopulation of cells targeted by *MLL*-fusion genes.

Unexpectedly, the expression level of induced *MLL-ENL* in MPD cells was higher under the *Tet1*-ablated condition. Since the induced *MLL-ENL* expression at the third plating of CD34(-) KSL-derived cells under the *Tet1*-ablated condition was comparable to that under the *Tet1*^+/+^ condition *in vitro*, the higher expression of induced *MLL-ENL in vivo* implied that the enrichment of cells that highly expressed *MLL-ENL* was more enhanced in association with the *Tet1*-ablation, in the process leading to MPD. The unexpected genetic effect, which may accelerate MPD under the *Tet1*-ablated condition, might reduce the effect of deceleration by loss of *Tet1*. However, in contrast to the finding that *Hoxa9* and *Meis1a* were markedly downregulated under *Tet1*-deficent conditions [14], our results showed that those genes were upregulated by *MLL-ENL* under *Tet1*-ablated conditions in the same way as *Tet1*^+/+^ conditions. In addition, irrespective of the genotypes, secondary BMT reproduced the MPD in similar latencies. In retrovirally *MLL-ENL*-immortalized CD34(+) KSL/MP cells, the conditional *Tet1*-ablation did not cause any significant changes in the clonogenicity or expression of critical downstream genes, although it should be noted that the ablation was incomplete presumably due to escape from recombination in a part of 4-OHT- treated cells, and/or the remaining expression of *Tet1* transcripts after the recombination. These results suggested that *Tet1* might not be essential for the *MLL-ENL*-mediated leukemogenesis, even after taking into consideration of the high expression of induced *MLL-ENL* in *Tet1*-ablated MPD cells.

The conditional *Tet1*-ablation was closely associated with very low *Evi1* expression in myeloid leukemogenesis by the induced *MLL-ENL in vivo*, but not in the myeloid immortalization assays *in vitro*. Considering the epigenetic function of Tet1 [8–10] and the recent finding that loss-of-function Tet leads to a localized increase in DNA methylation [30], further analyses, such as comprehensive profiling of the methylation status of regulatory genomic regions of *Evi1* [31], may be needed in the future. Since MLL-ENL upregulates *EVI1*/*Evi1* through promoter binding [32] and epigenetic regulation by histone modifications [33], epigenomic analyses regarding *Evi1* may be also helpful. Another hypothesis is that *Tet1*-ablation might lead to leukemic initiation by *MLL-ENL* more efficiently in relatively mature progenitor cells with low expression of *Evi-1*, since *MLL*-fusion genes upregulate *Evi1* in KSL cells more strongly than in myeloid progenitor cells [32,34,35]. Thus, further analyses including hematopoietic stem/progenitor-specific ablation of *Tet1* will be also needed to test this hypothesis.

In conclusion, to examine the role of *Tet1* in the myeloid leukemogenesis more precisely, we generated conditional *Tet1*-knockout mice, and constructed a mouse model enabling leukemic initiation, in combination with inducible *MLL-ENL* transgenic mice. The phenotypes of leukemic transformation by *MLL-ENL* were not critically affected by conditional *Tet1*-ablation. However, the low *Evi1* expression in MPD cells under the *Tet1*-ablated condition may be associated with a role of Tet1 in leukemic initiation by *MLL-ENL*. Furthermore, Tet1 was dispensable in leukemic maintenance in secondary BMT, suggesting that the role of Tet1 may not be essential for, at least, *MLL-ENL*-mediated myeloid leukemogenesis. Also, our results suggested that the therapeutic application of Tet1 inhibition in AML may need careful assessment.

## Supporting information

S1 Checklist ARRIVE guidelines checklist. (PDF)

S1 Fig The proportion of myeloid, B-, and T lymphoid cells in peripheral blood from conditionally *Tet1*-ablated mice. The blood samples were measured just before (0), 4, 8, and 12 weeks after tamoxifen treatment as shown in Fig 2A (n = 5 per each (the same mice as used in Fig 2A)). Bar graphs show the mean and SD of data combined from two independent experiments. n.s., not significant. (determined by two-way repeated measures ANOVA followed by Shaffer’s modified sequentially rejective Bonferroni procedure).
(TIF)

S2 Fig Assessment of hematopoiesis derived from conditionally *Tet1*-ablated bone marrow cells. (A) The experimental strategy for the hematological assessment of BM chimera mice where bone marrow (BM) cells were replaced with *CreER*;*Tet1*^fl/fl^ or *Tet1*^fl/fl^ BM cells (from one mouse per each genotype (total 2)) in bone marrow transplantation. Wt, wild-type; Tm, tamoxifen; ip, intraperitoneal injection; PB, peripheral blood. (B) Time course of measurement of the white blood cells (WBCs) counts and proportion of myeloid, B-, and T lymphoid cells in peripheral blood just before (0) and at the indicated number of weeks after tamoxifen treatment (n = 5 per each (total 10)). **p<0.005; n.s., not significant. (determined by two-tailed unpaired *t*-tests).
(TIF)

S3 Fig Expression of *Dnmt* genes in the conditionally *Tet1*-ablated cells that were retrovirally immortalized by *MLL-ENL* beforehand. The expression levels of *Dnmt1*, *Dnmt3a*, and *Dnmt3* assessed by RT-qPCR in the *CreER*;*Tet1*^fl/fl^ CD34(+) KSL/MP-derived cells retrovirally immortalized by *MLL-ENL* with treatment of 4-OHT or vehicle control (ethanol, EtOH). Bar graphs show the mean and SD of three independent experiments. n.s., not significant. (determined by two-tailed unpaired *t*-tests).
(TIF)

S4 Fig Expression of *Dnmt1* and *Dnmt3a* genes in myeloid immortalization assays by the induction of Tg-*ME*-derived *MLL-ENL* under simultaneous *Tet1*-ablation. The expression levels of *Dnmt1* and *Dnmt3a* in the cells harvested at the end of the first (1R) and the third (3R, only in CD34(-) KSL-derived cells) rounds of plating, as assessed by RT-qPCR. Bar graphs show the mean and SD of three independent experiments. *p<0.05; n.s., not significant; †, not significant in comparison of the other combinations. The p-values for the expression levels of the genes tested were determined by one-way ANOVA followed by Tukey-Kramer tests (CD34(+) and MP), or a combination of one-way ANOVA followed by Tukey-Kramer tests (1R) and two-way repeated measures ANOVA followed by Shaffer’s modified sequentially rejective Bonferroni procedure (*CreER*; *Tet1*^fl/fl^; Tg-*ME* and *CreER*; *Tet1*^+/+^; Tg-*ME*) adjusted using the Holm-Bonferroni correction (CD34(-) KSL).
(TIF)

S5 Fig The leukemogenesis by induction of Tg-*ME*-derived *MLL-ENL* under simultaneous *Tet1* ablation, compared with the counterpart controls. (A) Chimerism analyses in peripheral blood cells (shown in Fig 5A) just before (0) and 4 weeks after tamoxifen treatment in the cohort #1. *P*-values were determined by two-way repeated measures ANOVA followed by Shaffer’s modified sequentially rejective Bonferroni procedure. (B) Survival curves of the mixed-bone marrow (BM) chimera mice (cohort #1) receiving *CreER*; *Tet1*^fl/fl^; Tg-*ME* (n = 13), *CreER*; *Tet1*^+/+^; Tg-*ME* (n = 8), *CreER*; *Tet1*^fl/fl^ (n = 4), and *Tet1*^fl/fl^ (n = 5) BM cells that were treated with tamoxifen. (C) The typical morphology of BM cells from the moribund mice that developed lethal MPD. Cells were stained with Wright-Giemsa. Magnification, 200×; scale bars, 50 μm. Bar graphs show mean with SD of data combined from two independent experiments in (A). n.s., not significant. (determined by two-way repeated measures ANOVA followed by Shaffer’s modified sequentially rejective Bonferroni procedure).
(TIF)

S6 Fig Expression profiling of *Dnmt* genes in the leukemogenesis assays. The expression levels of *Dnmt1*, *Dnmt3a*, and *Dnmt3b* in BM cells from the moribund mice developing lethal MPD in the leukemogenesis assays under *Tet1*-ablated (*Tet1*^Δ/Δ^) and *Tet1*^+/+^-conditions, as assessed by RT-qPCR. Data are shown in box and whisker plots. n.s., not significant. (determined by two-tailed unpaired *t*-tests).
(TIF)

S1 Raw images Uncropped images for gels and Southern/Northern blots. (TIF)

S2 Raw images Uncropped images for gels and dot blots. (TIF)
