# Supplementary figures and images for: Tet1 is not required for myeloid leukemogenesis by MLL-ENL in novel mouse models
Source: PLoS One. 2021 Mar 11;16(3):e0248425. doi: 10.1371/journal.pone.0248425 (PMC7951824; doi:10.1371/journal.pone.0248425)

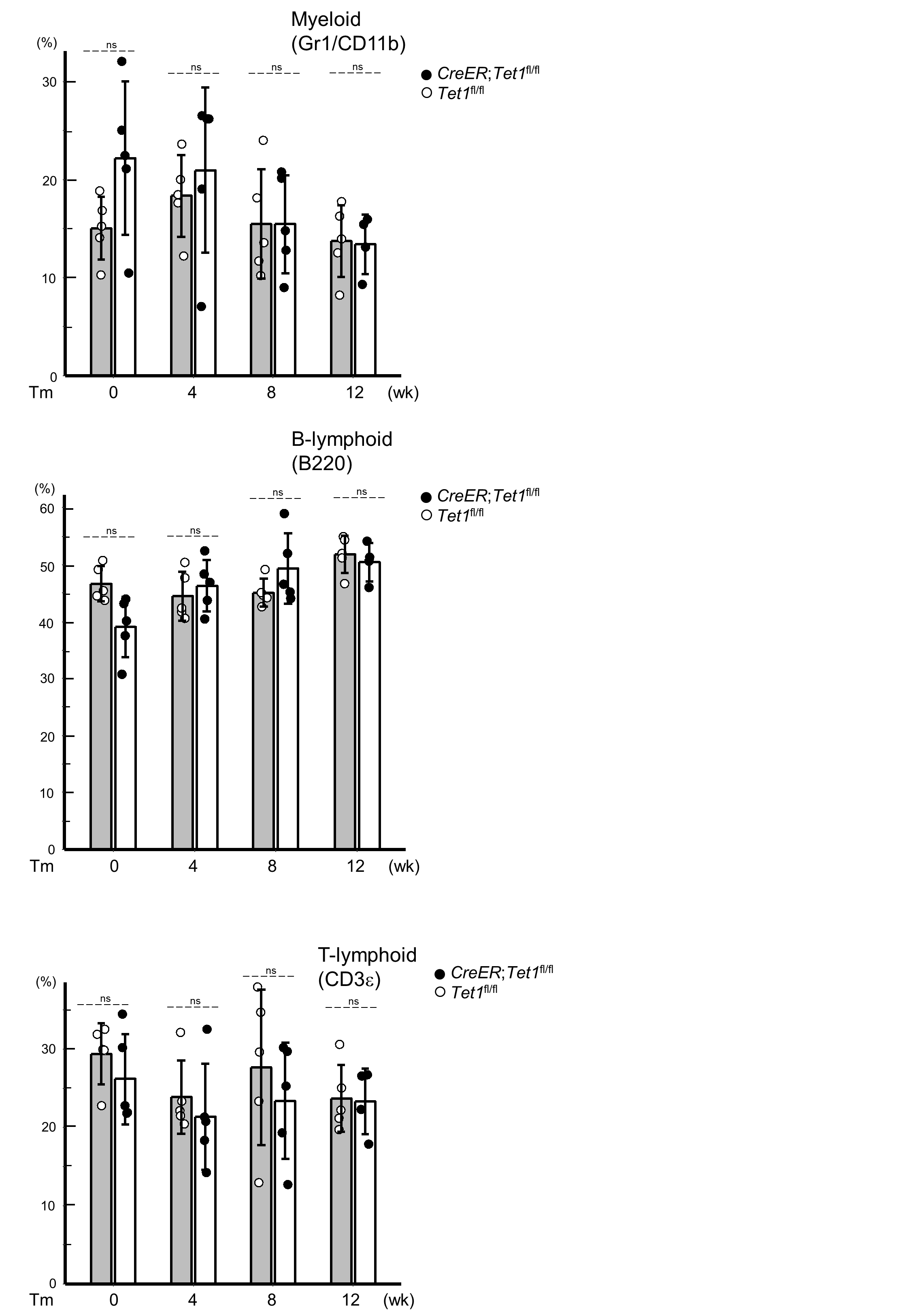

Supplement: S1 Fig — The blood samples were measured just before (0), 4, 8, and 12 weeks after tamoxifen treatment as shown in Fig 2A (n = 5 per each (the same mice as used in Fig 2A)). Bar graphs show the mean and SD of data combined from two independent experiments. n.s., not significant. (determined by two-way repeated measures ANOVA followed by Shaffer’s modified sequentially rejective Bonferroni procedure). (TIF) [file pone.0248425.s002.TIF]

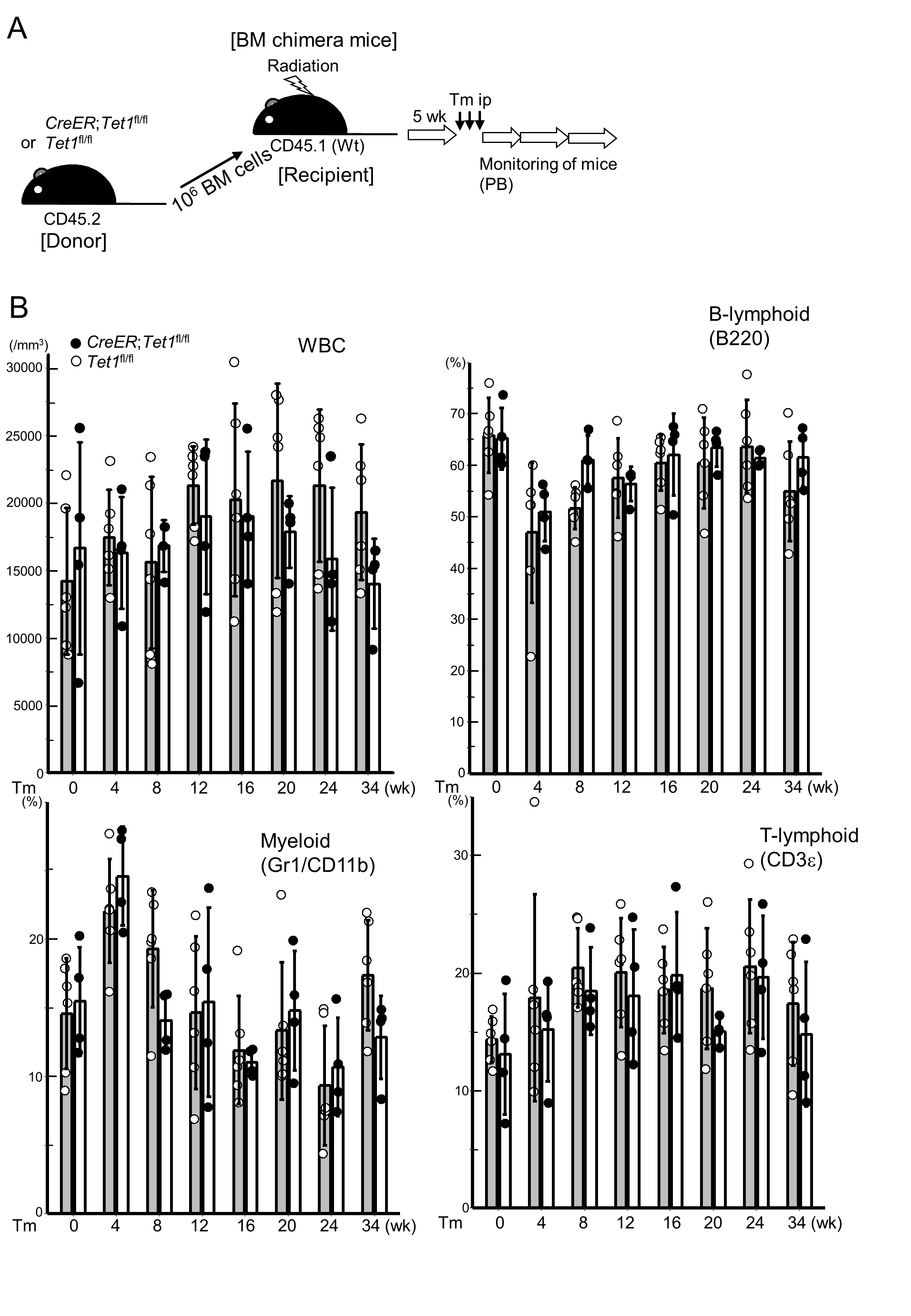

Supplement: S2 Fig — (A) The experimental strategy for the hematological assessment of BM chimera mice where bone marrow (BM) cells were replaced with CreER;Tet1fl/fl or Tet1fl/fl BM cells (from one mouse per each genotype (total 2)) in bone marrow transplantation. Wt, wild-type; Tm, tamoxifen; ip, intraperitoneal injection; PB, peripheral blood. (B) Time course of measurement of the white blood cells (WBCs) counts and proportion of myeloid, B-, and T lymphoid cells in peripheral blood just before (0) and at the indicated number of weeks after tamoxifen treatment (n = 5 per each (total 10)). **p<0.005; n.s., not significant. (determined by two-tailed unpaired t-tests). (TIF) [file pone.0248425.s003.TIF]

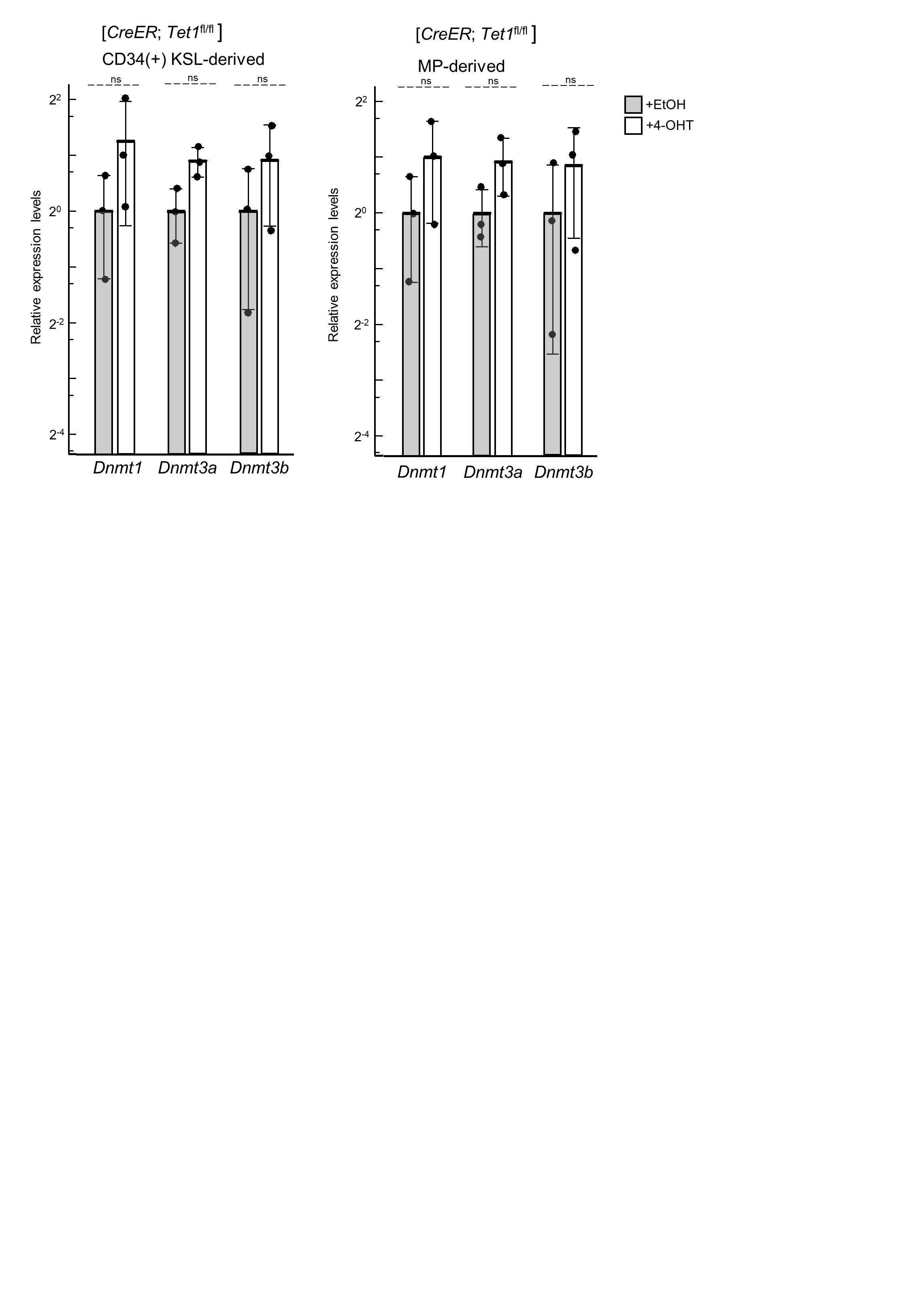

Supplement: S3 Fig — The expression levels of Dnmt1, Dnmt3a, and Dnmt3 assessed by RT-qPCR in the CreER;Tet1fl/fl CD34(+) KSL/MP-derived cells retrovirally immortalized by MLL-ENL with treatment of 4-OHT or vehicle control (ethanol, EtOH). Bar graphs show the mean and SD of three independent experiments. n.s., not significant. (determined by two-tailed unpaired t-tests). (TIF) [file pone.0248425.s004.TIF]

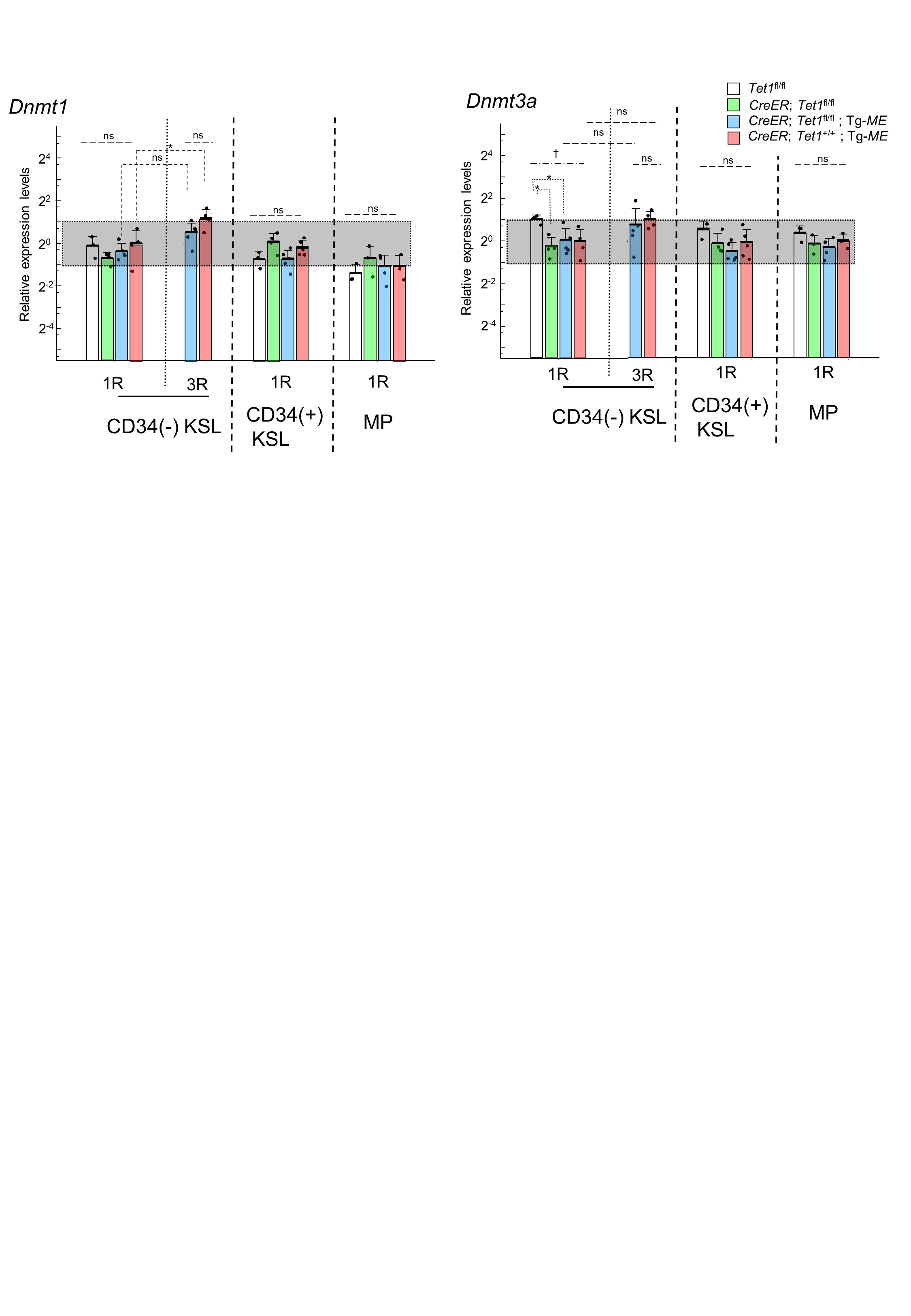

Supplement: S4 Fig — The expression levels of Dnmt1 and Dnmt3a in the cells harvested at the end of the first (1R) and the third (3R, only in CD34(-) KSL-derived cells) rounds of plating, as assessed by RT-qPCR. Bar graphs show the mean and SD of three independent experiments. *p<0.05; n.s., not significant; †, not significant in comparison of the other combinations. The p-values for the expression levels of the genes tested were determined by one-way ANOVA followed by Tukey-Kramer tests (CD34(+) and MP), or a combination of one-way ANOVA followed by Tukey-Kramer tests (1R) and two-way repeated measures ANOVA followed by Shaffer’s modified sequentially rejective Bonferroni procedure (CreER; Tet1fl/fl; Tg-ME and CreER; Tet1+/+; Tg-ME) adjusted using the Holm-Bonferroni correction (CD34(-) KSL). (TIF) [file pone.0248425.s005.TIF]

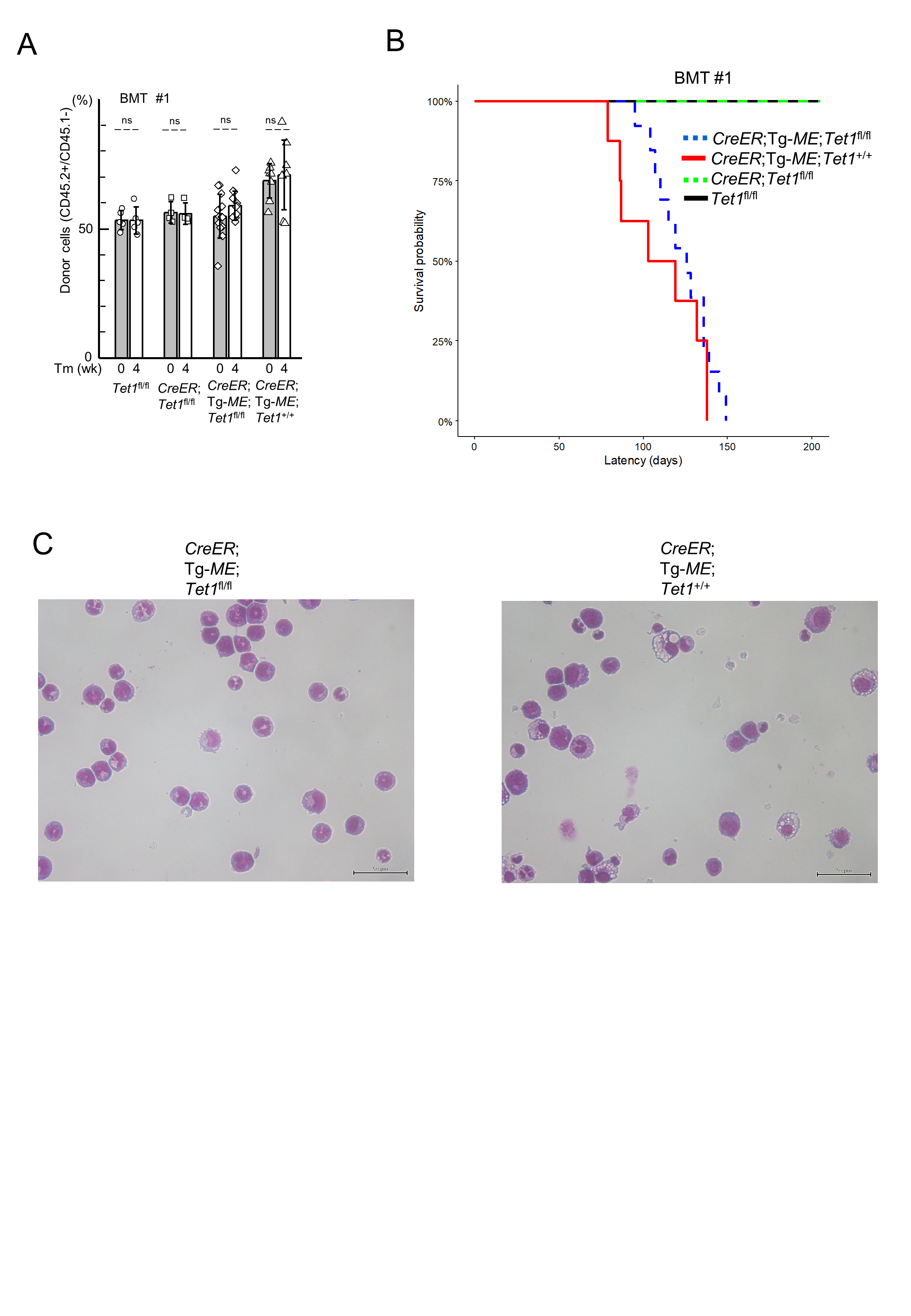

Supplement: S5 Fig — (A) Chimerism analyses in peripheral blood cells (shown in Fig 5A) just before (0) and 4 weeks after tamoxifen treatment in the cohort #1. P-values were determined by two-way repeated measures ANOVA followed by Shaffer’s modified sequentially rejective Bonferroni procedure. (B) Survival curves of the mixed-bone marrow (BM) chimera mice (cohort #1) receiving CreER; Tet1fl/fl; Tg-ME (n = 13), CreER; Tet1+/+; Tg-ME (n = 8), CreER; Tet1fl/fl (n = 4), and Tet1fl/fl (n = 5) BM cells that were treated with tamoxifen. (C) The typical morphology of BM cells from the moribund mice that developed lethal MPD. Cells were stained with Wright-Giemsa. Magnification, 200×; scale bars, 50 μm. Bar graphs show mean with SD of data combined from two independent experiments in (A). n.s., not significant. (determined by two-way repeated measures ANOVA followed by Shaffer’s modified sequentially rejective Bonferroni procedure). (TIF) [file pone.0248425.s006.TIF]

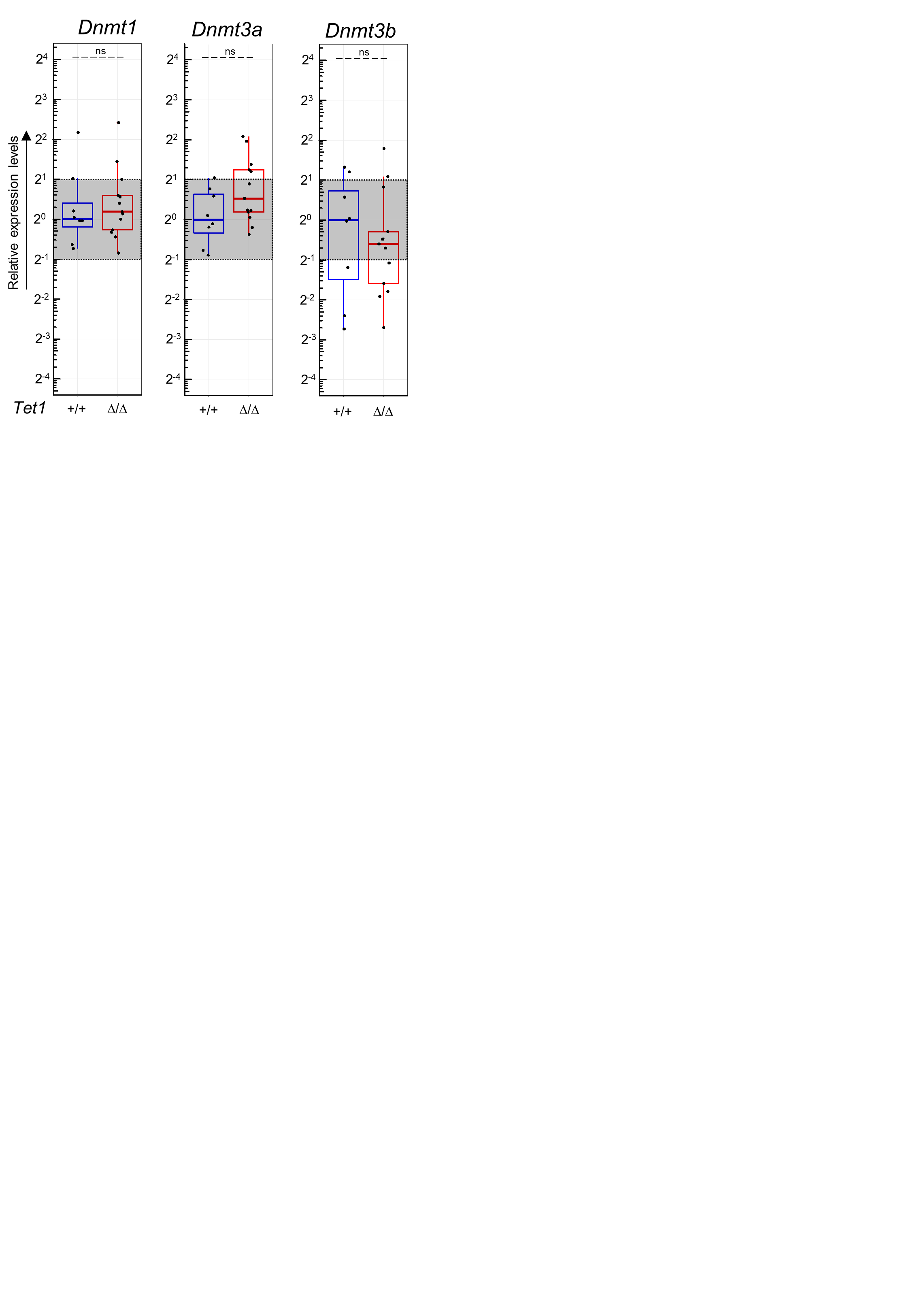

Supplement: S6 Fig — The expression levels of Dnmt1, Dnmt3a, and Dnmt3b in BM cells from the moribund mice developing lethal MPD in the leukemogenesis assays under Tet1-ablated (Tet1Δ/Δ) and Tet1+/+-conditions, as assessed by RT-qPCR. Data are shown in box and whisker plots. n.s., not significant. (determined by two-tailed unpaired t-tests). (TIF) [file pone.0248425.s007.TIF]

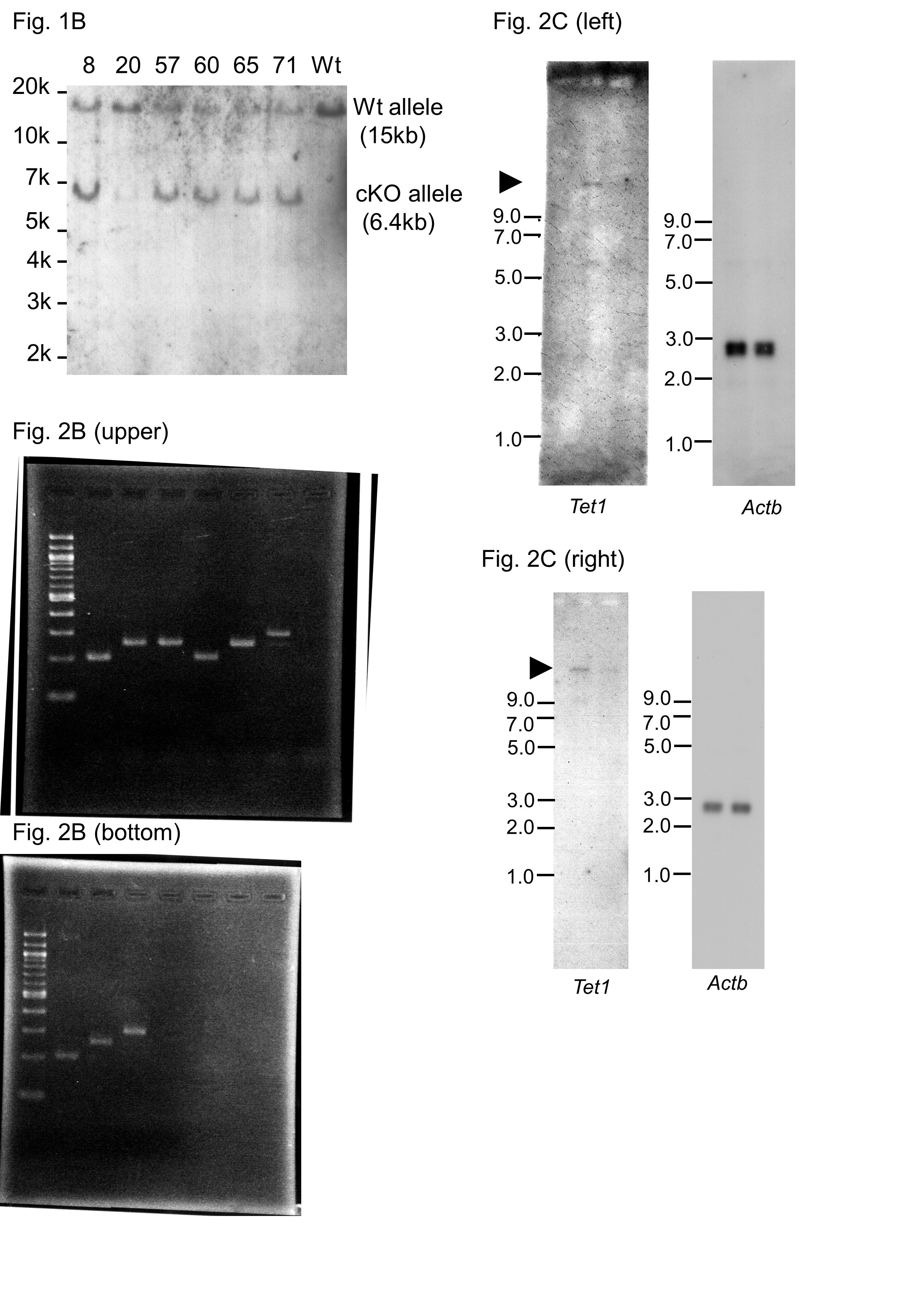

Supplement: S1 Raw images — (TIF) [file pone.0248425.s008.TIF]

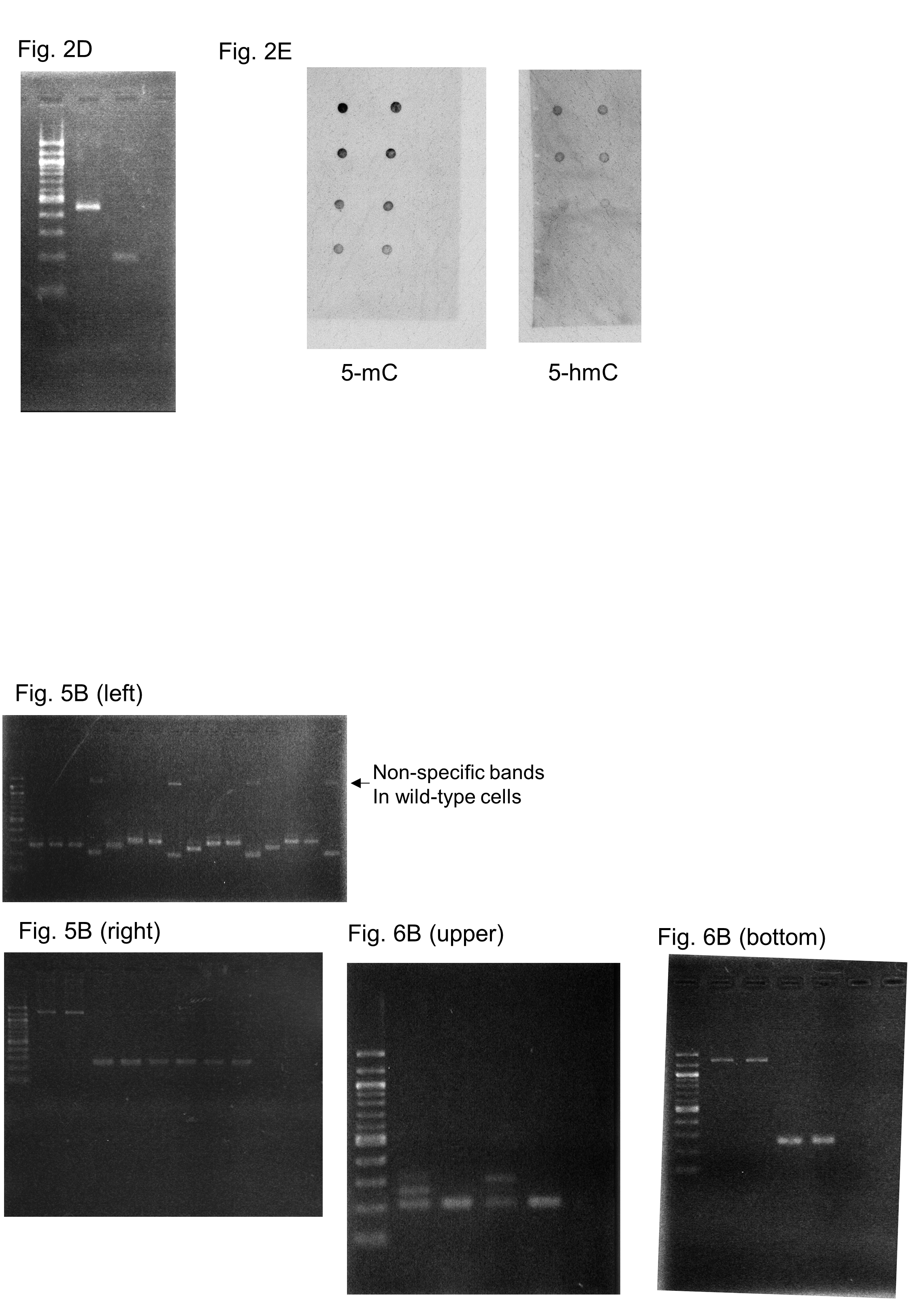

Supplement: S2 Raw images — (TIF) [file pone.0248425.s009.TIF]
